# Defining melanoma combination therapies that provide senolytic sensitivity in human melanoma cells

**DOI:** 10.3389/fcell.2024.1368711

**Published:** 2024-06-14

**Authors:** Daméhan Tchelougou, Nicolas Malaquin, Guillaume B. Cardin, Jordan Desmul, Simon Turcotte, Francis Rodier

**Affiliations:** ^1^ Centre de Recherche du Centre Hospitalier de l’Université de Montréal (CRCHUM) et Institut du Cancer de Montréal, Montreal, QC, Canada; ^2^ Département de chirurgie, Université de Montréal, Montreal, QC, Canada; ^3^ Département de Radiologie, Radio-oncologie et médicine nucléaire, Université de Montréal, Montreal, QC, Canada

**Keywords:** senescence, senescence-like, Bcl2/BclxL inhibitors, senolytic, BRAFi, MEKi, melanoma

## Abstract

Malignant Melanoma that resists immunotherapy remains the deadliest form of skin cancer owing to poor clinically lasting responses. Alternative like genotoxic or targeted chemotherapy trigger various cancer cell fates after treatment including cell death and senescence. Senescent cells can be eliminated using senolytic drugs and we hypothesize that the targeted elimination of therapy-induced senescent melanoma cells could complement both conventional and immunotherapies. We utilized a panel of cells representing diverse mutational background relevant to melanoma and found that they developed distinct senescent phenotypes in response to treatment. A genotoxic combination therapy of carboplatin-paclitaxel or irradiation triggered a mixed response of cell death and senescence, irrespective of BRAF mutation profiles. DNA damage-induced senescent melanoma cells exhibited morphological changes, residual DNA damage, and increased senescence-associated secretory phenotype (SASP). In contrast, dual targeted inhibition of Braf and Mek triggered a different mixed cell fate response including senescent-like and persister cells. While persister cells could reproliferate, senescent-like cells were stably arrested, but without detectable DNA damage and senescence-associated secretory phenotype. To assess the sensitivity to senolytics we employed a novel real-time imaging-based death assay and observed that Bcl2/Bcl-XL inhibitors and piperlongumine were effective in promoting death of carboplatin-paclitaxel and irradiation-induced senescent melanoma cells, while the mixed persister cells and senescent-like cells resulting from Braf-Mek inhibition remained unresponsive. Interestingly, a direct synergy between Bcl2/Bcl-XL inhibitors and Braf-Mek inhibitors was observed when used out of the context of senescence. Overall, we highlight diverse hallmarks of melanoma senescent states and provide evidence of context-dependent senotherapeutics that could reduce treatment resistance while also discussing the limitations of this strategy in human melanoma cells.

## 1 Introduction

Despite the revolutionary impact of immunotherapy, malignant melanomas remain the primary cause of skin cancer deaths worldwide (Bray et al., 2018; Siegel et al., 2020; Ferlay et al., 2021). BRAF and NRAS mutations in the MAPK pathway are common in about 50% and 25% of melanoma patients, respectively (Davies et al., 2002; Edlundh-Rose et al., 2006). Targeted therapies, like MEK and BRAF inhibitors, offer significant benefits, but the majority of patients eventually develop drug resistance and experience disease relapse (Perez-Lorenzo and Zheng, 2012).

Melanoma often harbors additional mutations in tumor suppressor genes such as TP53 (p53) and CDKN2A (p16) (Hodis et al., 2012; Shain and Bastian, 2016), which play crucial roles in regulating cellular senescence. Despite the remarkable clinical efficacy of immune checkpoint therapies, only about 50% of patients achieve lasting responses (Sharma and Allison, 2015; Havel et al., 2019). Consequently, there’s a significant unmet need for effective treatment for malignant melanoma, particularly for patients resistant to standard therapies (Chapman et al., 2011; Flaherty et al., 2012).

Standard cancer treatments like chemotherapy and irradiation induce various responses in melanoma cells, including cellular senescence (Mhaidat et al., 2007; Haferkamp et al., 2013; Jost et al., 2021). Cellular senescence was initially observed *in vitro* in primary cells undergoing extensive culture and replicative exhaustion linked to telomere shortening (Hayflick, 1965) and is primarily characterized by a stable proliferation arrest. Therapy-induced senescence has emerged as a common cell fate in response to cancer treatment, showing potential for therapeutic intervention (Salama et al., 2014; Fleury et al., 2019; Chakrabarty et al., 2021). Recent studies also indicate senescent cells can contribute to treatment resistance and secondary cancers development in various malignancies, including melanoma (Guillon et al., 2019; Chakrabarty et al., 2021; Thompson et al., 2021).

Preclinical evidence indicates that removing senescent cells, genetically or with drugs, can slow disease progression and improve overall health (Baar et al., 2017; Xu et al., 2018; Yousefzadeh et al., 2018; Mylonas et al., 2021). Also, targeting senescent cancer cells using senotherapy (senolytic drugs) have been shown to reduce side effects of senescence-inducing treatments (Fleury et al., 2019; Wang et al., 2019; Jochems et al., 2021). Therefore, senotherapy holds promising therapeutic potential.

Recently, our team and others have demonstrated the sensitivity of ovarian, breast, sarcoma, prostate, pancreatic ductal adenocarcinoma, glioma and liver cells to senolytics through the selective elimination of senescent cells generated by primary therapies, employing a one-two-punch combinatorial therapeutic approach (Fleury et al., 2019; Wang et al., 2019; Balakrishnan et al., 2020; Malaquin et al., 2020; Lafontaine et al., 2021; Jaber et al., 2023). Importantly, these studies have investigated multiple cancer treatment modalities and panels of potential senolytic drugs, revealing the context-dependent nature of senescence induction and senolytic sensitivity. Several studies have provided valuable insights into the mechanisms and implications of senescence in melanoma development, progression, and therapy resistance (Giuliano et al., 2011; Maertens et al., 2013; Thompson et al., 2021). Noteworthy studies have explored the senescence-associated molecular changes, signaling pathways, and interactions with the tumor microenvironment in solid tumors (Oubaha et al., 2016; Milanovic et al., 2018). Furthermore, other studies have investigated the impact of senescence on immunotherapy response and the potential of senolytic drugs to enhance therapeutic outcomes in solid tumors (Ruscetti et al., 2021). Collectively, these studies have underscored the importance of senescence in melanoma as a potential actionable target for therapeutic strategies.

In recent studies focusing on senescence in melanoma, researchers have made significant contributions to our understanding of the implications of senescence in this disease. Liang et al. identified distinct senescence-associated gene expression signatures in melanoma samples, correlating with disease progression and patient outcomes (Liang et al., 2023). Others explored the reciprocal interactions between senescent melanoma cells and the tumor microenvironment, shaping the immune landscape (Lin et al., 2023), and the endothelial senescence signature serving as prognostic markers for survival and immune response prediction (Wu et al., 2023).

Previously, authors demonstrated the potential of targeting the senescence-associated secretory phenotype (SASP) to enhance the response of melanoma cells to immunotherapy (Milanovic et al., 2018). Additionally, others highlighted the role of therapy-induced senescence in promoting drug resistance and found that the presence of senescence markers, such as p16 and senescence-associated βeta-galactosidase (SA-β-gal), correlated with poor prognosis and decreased overall survival in melanoma patients (Thompson et al., 2021). These studies collectively underscore the importance of investigating senescence in melanoma and provide valuable insights for developing novel therapeutic strategies.

In this study, our objective was to comprehensively assess the diverse cell fate outcomes induced by clinically relevant therapies in melanoma and investigate the potential of a combination senolytic approach to enhance therapeutic responses in specific contexts. Our findings reveal a wide spectrum of melanoma therapy-induced cell fate decisions and demonstrated that treatments triggering persistent DNA damage in senescent melanoma cells are amenable to a senolytic strategy involving Bcl2/Bcl-XL inhibitors.

## 2 Material and methods

### 2.1 Cell lines and cell culture

Human melanoma cancer cell lines used in this study: Mel-SK23 which expresses wild-type BRAF, Mel-1102 carrying NRASQ61 K mutation, and two cell lines with BRAFV600E mutation (Mel-624.38 and Mel-526). These cell lines were originally obtained from the National Cancer Institute (NCI), NIH, Bethesda, MD United States (Topalian et al., 1989) and were generously provided by Dr. Réjean Lapointe at the Centre de Recherche du Centre Hospitalier de l'Université de Montréal (CRCHUM), Canada. All cell lines were routinely screened and confirmed to be negative for *mycoplasma*. Cells were cultured in RPMI medium with 8% fetal bovine serum, 100 I.U ml^-1^ penicillin, 100 μg mL^-1^ streptomycin, and 2 mM ml^-1^ L-glutamine (all obtained from Wisent, QC, Canada).

### 2.2 Viruses and infections

H2B-GFP lentiviruses were produced as described previously (Fleury et al., 2019) and viral titers were adjusted to achieve ∼90% infectivity (Rodier et al., 2011). Infections were followed 48 h later by hygromycin selection (200 μg/mL for 6 days) and stable cells were used in experiments. The generation of H2B-GFP-infected cells and related methodologies can be found in our previous publication (Fleury et al., 2019).

### 2.3 Drug treatments and irradiation

ABT-263 (Navitoclax) was from APExBIO (Houston, TX, United States of America). A-1155463 (S7800) was from Selleckchem (Houston, TX, United States of America). Piperlongumine (1919) was from BioVision (Milpitas, CA, United States of America). ABT-199 (Venetoclax) and Dabrafenib were from Cayman Chemical (Ann Arbor, MI, United States of America). Trametinib was from Toronto Research Chemicals (North York, ON, CA). Carboplatin and paclitaxel were from Accord Healthcare (Kirkland, QC, CA). Drugs were first dissolved in 100% dimethyl sulfoxide (DMSO) and then further diluted in complete culture media for each experiment. For treatments with senolytic, we first conducted a dose-response assay to determine the half-maximal inhibitory concentration (IC50) for each senolytic in each melanoma cell line (Supplementary Figure S1A). The final concentrations used in subsequent assays were selected based on this dosage combined to established precedents (Fleury et al., 2019; Malaquin et al., 2020). The drugs were added to the cell culture 24 h after seeding, and BMi treatment was refreshed every 3 days throughout all experiments. The selected concentrations for both BMi (50 nM and 5 nM), or carboplatin (10 µM) and paclitaxel (30 nM) in combination treatment (administered over a period of 24 h), were determined based on clinically relevant doses that are reflective of plasma levels observed in treated patients (Huizing et al., 1997; Ozols et al., 2003; Puszkiel et al., 2019; Goldwirt et al., 2021). After treatment, cells were washed with PBS (twice) and provided with fresh complete culture media. Irradiation was performed using Gammacell^®^ 3000 irradiator Elan at a dose rate of 0.75 Gy/min for a total dose of 10 Gy followed by a fresh media change.

### 2.4 Real-time cell proliferation phase-contrast imaging assay

For live cell proliferation assessment in 96-well, 1000 cells/well were seeded for Mel-SK23, 3000 cells/well were seeded for Mel-1102, and 1500 cells/well were seeded for Mel-624.38 and Mel-526 (all expressing H2B-GFP). Cells were either irradiated (10 Gy) before seeding, or incubated with DMSO (control), or Braf and Mek inhibitors (Dabrafenib + Trametinb at 50nM+5 nM) or carboplatin and paclitaxel (10µM + 30 nM) at different times. IncuCyte^™^ Live-Cell Imaging System (IncuCyte HD) was used to image cell number by phase contrast and fluorescence. Frames were captured every 8 h (×10 objective). Proliferation data were analyzed by using IncuCyte^™^ S3 software based on green element count (H2B-GFP cell nuclei) or cell confluency and proliferation curves were plotted by GraphPad Prism 9.0 software (GraphPad Inc., San Diego, CA). Each experiment was performed in triplicate and repeated three times.

### 2.5 Real-time cell death imaging and specific death assays

Propidium iodide (PI) fluorescently label dead cells based on the loss of cell membrane integrity (PI is normally a cell impermeant DNA binding dye (Belloc et al., 1994). PI (Sigma Aldrich, Saint-Louis, United States) was added to the culture media at a concentration of 0,5 μg/mL, and cells imaged as for the real-time proliferation assay. Frames were captured at 8 h intervals from two separate regions per well using ×10 objective. The IncuCyte^™^ S3 software was used to quantify the percent of dead cells by scoring PI positive fluorescently labeled red nuclei against total green H2B-GFP cell nuclei. Graph were plotted using GraphPad Prism 9 (GraphPad Inc., San Diego, CA). For quantification of specific death caused by senolytic combinations we adapted a chromium-51 release assay formula (Wallace et al., 2004). We normalized the death data using two strategies, first by removing the baseline level of cell death in each condition (from the matched control), second by creating a 100% death measurement using complete cell lysis with a short Triton treatment at concentration of 0,01% in the culture media (TritonTM X-100 solution, Sigma Aldrich, Saint-Louis, United States) (Cummings and Schnellmann, 2004). To further ascertain that we measured the additional cell death caused by the senolytic treatment, prior to senolytics drugs and PI addition, melanoma cells (treated or untreated) were washed with PBS to remove floating cells. Each experimental condition was performed in triplicate and repeated at least two times.

$$Specific\;death\;\left(percent\right)=\left(\frac{Experimental\;death-Spontaneous\;death}{Maximum\;death-\;Spontaneous\;death}\right)X\;100$$

Experimental death = (PI+/H2B-GFP+)/(H2B-GFP+) In each experimental condition.Spontaneous death = (PI+/H2B-GFP+)/(H2B-GFP+) In control (DMSO) condition.Maximum death = (PI+/H2B-GFP+)/(H2B-GFP+) In Triton treated condition.Each experiment was performed in triplicate and repeated at least 2 times.

### 2.6 Clonogenic assays

Cells were seeded in a 6-well plate at a density of 500 cells (Mel-SK23), 2000 cells (Mel-1102) or 1000 cells (Mel-624.38 and Mel-526) per well. The media was removed and replaced with complete media (RPMI 8%FBS) containing Braf and Mek inhibitors alone or in combination at indicated concentrations. Cells were treated for 15 days and analyzed or released for an additional 15 days in a drug-free medium and analyzed. Cells were fixed and colored with a mix of 50% v/v methanol and 0.5% m/v of crystal violet (Sigma-Aldrich Inc., St. Louis, MO). Colonies were counted under a stereomicroscope at a ×2 magnification and reported as a percentage of control. Each experiment was performed in duplicate and repeated 3 times.

### 2.7 Immunofluorescence and pulsed DNA synthesis detection

Cells were seeded in 8-wells chamber slides (Life Sciences, Corning, NY, United States) and allowed to adhere for 24 h before exposition to treatments. Cells were fixed for 5 min in formalin at room temperature (RT) and permeabilized in 0.25% Triton in phosphate-buffered saline (PBS) for 10 min. Slides were blocked for 1 h in PBS containing 1% bovine serum albumin (BSA) and 4% donkey serum. Primary antibodies (γH2AX and 53BP1) diluted (1/2500) in blocking buffer were added in each well and slides were incubated overnight at 4 °C. Cells were washed (PBS) and incubated with secondary antibodies (dilution 1/5000) for 1 h at RT, then washed again.

To detect DNA synthesis EdU (5-ethynyl-2′-deoxyuridine; 10 μM, Invitrogen) was added to the medium and incubated for 24 h from days 8–9 post-treatment. Cells were washed three times with TBS and fixed with 10% formalin for 5 min. EdU fluorescence staining was assessed using the Click-iT^®^ EdU Alexa Fluor^®^ 488 Imaging Kit (Invitrogen).

For immunofluorescence and EDU coverslips were mounted onto slides using Prolong^®^ Gold antifade reagent with DAPI (Life Technologies Inc.). Images were obtained using a Zeiss microscope (Zeiss AxioObserver Z1, Carl Zeiss, Jena, Germany). An automated analysis software from Zeiss (AxioVision^™^, Carl Zeiss) was used to count DNA damage foci to calculate the average number of foci per nucleus. The fold change was calculated as the ratio between percentages of γH2AX or 53BP1 nuclear foci in treated *versus* control (nontreated) cells. DNA damage positive cells percentage was calculated relative to the DAPI staining (total nuclei count). γH2AX and 53BP1 foci were quantified in more than 50 nuclei from three different fields of each chamber. For DNA synthesis evaluation EdU positive cells were similarly counted and reported to total nuclei (DAPI).

### 2.8 Analysis of cell cycle by flow cytometry

Cells were seeded in 6-well plates and treated 24 h after seeding. At each indicated time, cells were trypsinized, washed with PBS and fixed in cold ethanol (70%) for 24 h. Cells were then washed with PBS and stained for 30 min at room temperature with a 25 μg/mL PI solution containing 100 μg/mL RNAse A. The PI fluorescence signal was detected using the Fortessa flow cytometer (BD Biosciences, Mississauga, ON) and analyzed with FlowJo™ v10.8 Software (BD Life Sciences).

### 2.9 SA-β-gal detection

We adapted the SA-β-Gal protocol used by Fleury et al. (Debacq-Chainiaux et al., 2009; Fleury et al., 2019). Briefly, cells were seeded in 6-well plates and treated 24 h after seeding. At the endpoint (day 9 post-treatment), cells were washed once with PBS, fixed with 10% formalin for 5 min, washed again with PBS, then incubated at 37 °C for 12–16 h (depending on the cell line) in a staining solution composed of 1 mg mL^−1^ 5-bromo-4-chloro-3-inolyl-β-galactosidase in DMSO (20 mg mL^−1^ stock), 5 mM potassium ferricyanide, 150 mM NaCl, 40 mM citric acid/sodium phosphate, and 2 mM MgCl_2_, at pH 6.0. Finally, cells were washed twice with PBS and at least four representative pictures per well were taken for quantification using EVOS™ FL Digital Inverted Fluorescence Microscope from Thermo Fisher Scientific (Carlsbad, CA, United States). Each experiment was performed in duplicate (6 well plate) and repeated 3 times.

### 2.10 Nuclear size measurement

Nuclear size measurements were conducted using the ImageJ software (https://imagej.nih.gov/ij/) to quantify nuclear area based on DAPI nuclear staining. This was applied to high-resolution images acquired from the EVOS™ FL Digital Inverted Fluorescence Microscope (Thermo Fisher Scientific).

### 2.11 Protein extraction and Western blot analysis

Cells were seeded in Petri dishes (100 mm) and allowed to adhere for 24 h before each treatment. At the indicated times, cells were lysed in mammalian protein extraction reagent (MPER, Thermo Fisher Scientific, Waltham, MA) containing a protease and phosphatase inhibitor cocktail (Sigma-Aldrich Inc., St. Louis, MO). After protein quantification (Pierce BCA Protein Assay Kit, Thermo Fisher Scientific), 15 μg of total protein were separated using stain-free 4%–15% gradient Tris-glycine SDS-polyacrylamide gels (Mini PROTEAN^®^ TGX Stain-Free^™^ Gels, Bio-Rad Laboratories, CA) and transferred onto PVDF membranes (Amersham Hybond, GE Healthcare Life Sciences, Mississauga, ON, Canada). Immunodetections were performed using enhanced chemiluminescence (Thermo Fisher Scientific) to detect peroxidase-conjugated secondary antibodies bound to primary antibodies. A ChemiDoc MP Imaging System (Bio-Rad Laboratories) was used to detect chemiluminescence. The stain-free technology (Bio-Rad Laboratories) was used to quantify protein loading in the gel/membrane. Immunoreactive band intensities were quantified using ImageJ software (https://imagej.nih.gov/ij/).

### 2.12 IL8 secretion and analysis of secreted SASP factors in conditioned medium

Conditioned media - CM were prepared by incubating cells in 200 µL RPMI complete medium (FBS 8%) per well using 96-well treated culture microplate (Falcon ^®^; #353072) for 48 h and stored at −80 °C until probed. Levels of IL-8 were assessed using ELISA (R&D Systems (IL-8 #DY208). The data were normalized to cell number and reported as concentration of secreted protein per mL per cell. We also analyzed the CM using multiplex ELISA. 40 secreted factors were screened utilizing the V-Plex human kit supplied by Meso Scale Discovery (MSD; #K15209D), in accordance with the protocol provided by the manufacturer. The data were normalized to cell number and reported as concentration of secreted protein per mL per cell or as log2 fold changes of secreted protein to appropriate control.

### 2.13 Antibodies

The following antibodies were used: phospho-histone γ-H2AX (clone JBW301, EMD Millipore, Temecula, CA) (dilution for immunofluorescence 1/2500); 53BP1 (clone 305, Novus Biologicals, Littleton) (dilution for immunofluorescence 1/2500). p53 (clone DO-1, Santa Cruz, 1/5000) ERK-1/2 and phospho-ERK-1/2, 1/2500, Cell Signaling, #4370S; phospho-Rb (Ser 807/811), Cell Signaling, 1/2500; phospho-p90-RSK, Cell Signaling, 1/2000; p21, BD, 1/2000; GAPDH, Cell Signaling, 1/2500; Tubulin-a, 1/5000, Cell Signaling, 3873P); Bcl-2, Santa Cruz, #A0807, 1/1000; Bcl-XL, Cell Signaling, #5446, 1/1000.

## 3 Results

### 3.1 Varied melanoma treatments trigger distinct cell fate phenotypes

**FIGURE 1:**
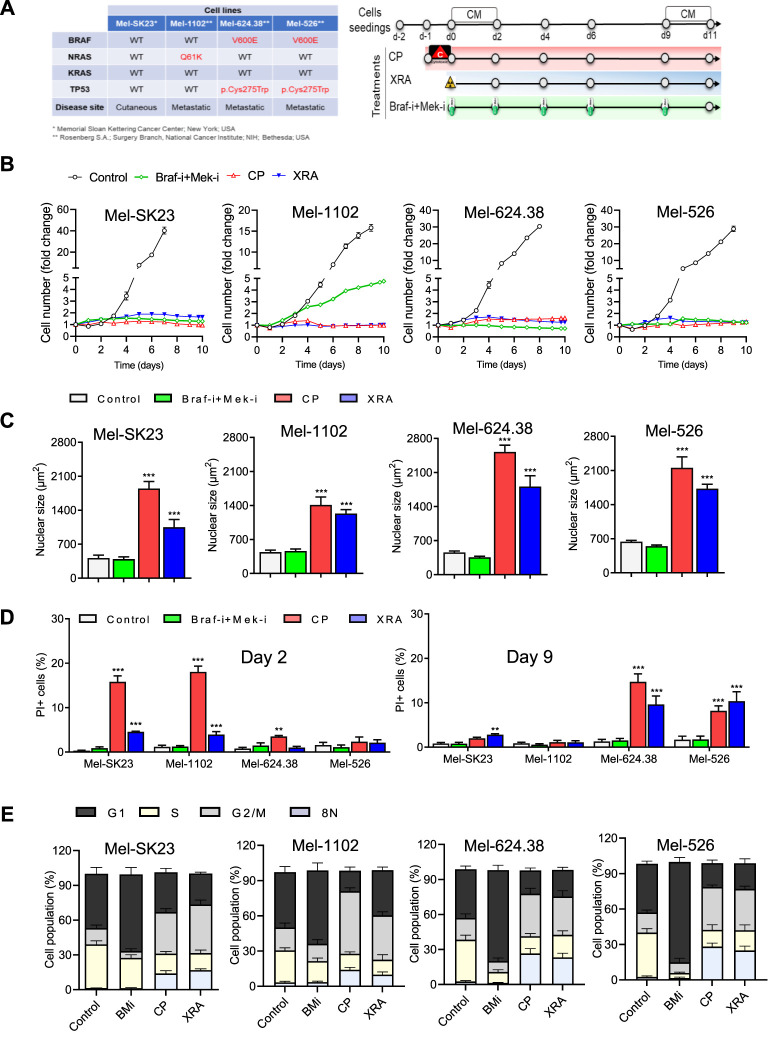
Varied cancer treatments trigger distinct cell fate phenotypes in melanoma cells. **(A)** The table on the left displays the genetic background of the selected melanoma cell lines, while the treatment timeline is presented on the right. **(B)** Proliferation curves of melanoma cells exposed to different treatments, including Dabrafenib (BRAF-i, 50 nM) + Trametinib (MEK-i, 5 nM, BRAF-i + MEK-i), Carboplatin plus Paclitaxel (CP, 10 µM + 30 nM respectively) or Radiation (XRA, 10 Gy). **(C)** The average nuclear size of H2B-GFP melanoma cells is compared between untreated cells and those treated for 6 days). **(D)** Quantification of cell death, determined by PI incorporation at day 2 and day 9 post-treatment using live imaging. **(E)** Cell cycle distribution of melanoma cells treated for 9 days compared to their respective controls. The data represents the mean ± SD of three independent experiments. Statistical significance is indicated by * for *p* < 0.05, ** for *p* < 0.01, and *** for *p* < 0.001, as determined by two-way ANOVA followed by Dunnett’s multiple comparisons test.

To assess the impact of diverse cancer therapies on melanoma cells, we used four human melanoma cell lines representing distinct clinical features (Figure 1A, left), including wild-type BRAF (Mel-SK23), NRASQ61 K mutation (Mel-1102), and BRAFV600E and TP53 mutations (Mel-624.38 and Mel-526). Lentiviral labeling with H2B-GFP enabled cell nucleus visualization, and cells were subjected to various therapies (Figure 1A, right), including Dabrafenib and Trametinib (BRAF and MEK inhibitors respectively). We monitored cell proliferation through live cell imaging (Supplementary Figures S1C–E).

Dabrafenib alone inhibited the proliferation of Braf V600E mutant melanoma cells, whereas Mel-SK23 and NRASQ61K Mel-1102 cells maintained their proliferative potential (Supplementary Figure S1C). Trametinib alone hindered the proliferation of all cell lines, with higher efficacy in Mel-624.38 and Mel-526 cells (Supplementary Figure S1D). The combination of BRAF and MEK inhibitors (BMi) halted the proliferation of Braf mutant cells (Supplementary Figures S1B, E). Alternatively, the NRASQ61 K mutated Mel-1102 cells exhibited a limited response to BRAF inhibition due to paradoxical ERK activation, a phenomenon where RAF inhibitors can induce the activation of ERK signaling in cells with upstream activating mutations such as NRAS (Supplementary Figure S1B) (Lai et al., 2022). Overall these findings confirm the genetic profile of MAP-kinase signaling in our melanoma cell line panel.

We then also exposed the panel of melanoma cells to conventional chemotherapies and radiotherapies, including a combination of carboplatin and paclitaxel (10 µM of Carboplatin +30 nM of Paclitaxel, referred to as CP) and ionizing radiation (10 Gy of X-ray, referred to as XRA). Remarkably, all melanoma cells, irrespective of their mutation status, exhibited substantial proliferation arrest after CP or XRA treatment (Figure 1B).

Cancer cells can respond to anti-cancer therapies in various ways, such as cell death, senescence, mitotic catastrophe, autophagy, or altered proliferation (Fu et al., 2021). We focused on melanoma cell fate decisions following exposure to CP, XRA, or BMi. Live cell imaging revealed that CP and XRA treatments led to nuclei enlargement within 2 days, suggesting increased ploidy and genome instability. Subsequent flow cytometry analysis identified G2/M accumulation and increased 8N polyploid cells after CP and XRA (Figure 1E). Conversely, BMi-treated cells demonstrated G1 phase accumulation and lacked these features (Supplementary Figures S1C, S2A, S1F). These results emphasize the distinct cellular responses triggered by different treatment modalities.

We then examined cell death in treated melanoma cells using real-time imaging with dynamic propidium iodide (PI) incorporation. CP and XRA induced some cell death, whereas BMi did not (Figure 1D). Notably, cell death occurred earlier in wild type p53 cells with CP and XRA treatment, while in mutated p53 cells it occurred later, indicating distinct responses based on p53 status.

### 3.2 Surviving melanoma cells exhibit a senescence phenotype following DNA damaging treatment

Following CP and XRA treatments, a proportion of melanoma cells underwent cell death, while surviving cells exhibited a senescent phenotype. The potential role of cellular senescence in mediating tumor microenvironment responses to cancer therapies led us to investigate senescence hallmarks in these cells.

**FIGURE 2:**
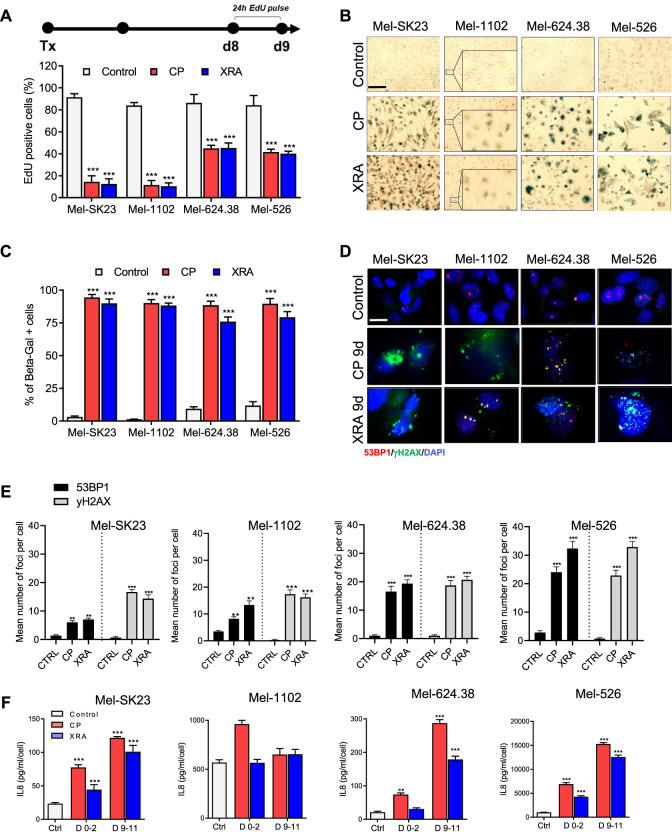
Surviving melanoma cells exhibit a senescence phenotype following genotoxic treatment. **(A)** Timeline illustrating the EdU pulse and the corresponding quantification of 24 h EdU-positive cells from day 8 to day 9 post-treatment. **(B,C)**: Representative images **(B)** and corresponding quantification **(C)** of SA-β-gal staining in melanoma cells, fixed 9 days after treatment with CP or XRA. **(D,E)**: Representative images **(D)** and quantification **(E)** of γH2AX and 53BP1 foci in the indicated cell lines, as determined through immunofluorescence. **(F)** Quantification of IL8 secretion, measured via ELISA, presented for the indicated cells during two different time frames: from day 0 to day 2 and from day 9 to day 11 post-treatments. The data represents the mean ± SD of three experiments, with statistical significance denoted by * for *p* < 0.05, ** for *p* < 0.01, and *** for *p* < 0.001, determined by two-way ANOVA followed by Dunnett’s multiple comparisons test.

We assessed the proliferative capacity of surviving cells through a 24-h Edu pulse assay, measuring DNA synthesis (Figure 2A top). After 9 days of CP and XRA treatments, Mel-SK23 and Mel-1102 cells displayed a significant reduction in Edu-positive cells (about 20% residual positivity), while Mel-624.38 and Mel-526 cells had around 40% of cells remaining Edu-positive (Figure 2A, Supplementary Figure S2B). Furthermore, cell cycle distribution analysis revealed a G2/M phase arrest following S phase, with increased aneuploidy (8N) and genome instability in p53-mutated melanoma cells compared to wild-type p53 cells.

Additionally, SA-β-Gal staining indicated a significant increase in beta-galactosidase activity in melanoma cells 9 days after CP and XRA treatment (Figures 2B, C). Immunofluorescence assays demonstrated the presence of persistent DNA double-strand break damage foci marked by 53BP1 and phosphorylated histone H2AX in these senescent cells (Figures 2D, E).

Three out of four cell lines also exhibited elevated levels of p21 proteins in p53 dependent (Supplementary Figures S4A–D) and p53 independent manner (Supplementary Figures S4G, H). Moreover, Mel-SK23 and Mel-1102 cells exhibited increase expression of both Bcl-2 and Bcl-XL at day 9 following CP treatment, while Mel-624.38 and Mel-526 did not (Supplementary Figures S4A, B). Together these observations highlight the presence of senescent cells following CP and XRA treatments.

Accumulated DNA damage response (DDR) foci and genome instability contribute to cellular senescence induction (Ghadaouia et al., 2021). The activation of NF-kappaB, crucial for upregulation of SASP cytokines (Elliott et al., 2001; Wang et al., 2017), is another consequence of DDR foci and genome instability. Considering the importance of the SASP factor IL8 in melanoma pathogenesis (Filimon et al., 2021), we analysed IL8 levels via ELISA and observed a time-dependent increase in secretion, except in the Mel-1102 cell line (NRASQ61 K). In general CP and XRA-treated cells exhibited four to over 10 times higher IL8 secretion compared to untreated cells (Figure 2F).

To take a broader look at the SASP secretome, we confirmed IL8 using a different multiplex ELISA analysis, which also included 38 additional secreted factors. Notably, this analysis revealed a significant elevation in the levels of several key cytokines and chemokines, including, but not limited to, IL-10, IFN-γ, IL-6, IP-10, MCP-1, VEGF and sICAM-1 in CP-treated melanoma cells (Supplementary Figures S2C–G). Together these results indicate that melanoma cells display a classical senescence phenotype in response to DNA damage-inducing treatments.

### 3.3 Long-term BMi elicit mixed responses of senescence-like and persister cells in Braf V600E melanoma cells

**FIGURE 3:**
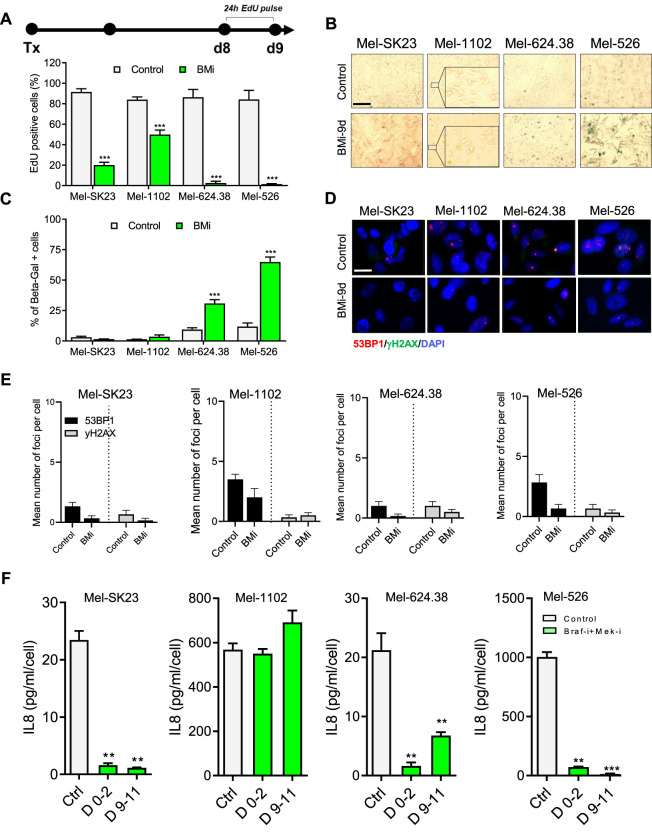
BMi trigger a senescence-like phenotype in Braf V600E melanoma cells. **(A)** Timeline illustrating the EdU pulse and the corresponding quantification of 24-h EdU-positive cells from day 8–9 following BMi treatment. **(B,C)** Representative images **(B)** and their respective quantification **(C)** of SA-β-gal staining in melanoma cells, fixed 9 days after BMi treatment. **(D,E)** Representative images **(D)** and quantification **(E)** of γH2AX and 53BP1 foci in indicated cell lines, analyzed via immunofluorescence. **(F)** Quantification of IL8 secretion, measured using ELISA, during two distinct time frames: from day 0 to day 2 and from day 9 to day 11 following BMi treatment in the indicated cells. Data represents the mean ± SD of three experiments, with statistical significance indicated by * for *p* < 0.05, ** for *p* < 0.01, and *** for *p* < 0.001, as determined by two-way ANOVA followed by Dunnett’s multiple comparisons test.

To assess senescence hallmarks following combined Braf and Mek inhibition, we continuously exposed melanoma cells to BMi for 9 days. In a 24-h EdU pulse experiment (Figure 3A top), we observed that wild-type Braf melanoma cells (Mel-SK23 and Mel-1102) had a relatively high frequency of EdU-positive cells (19% and 50% respectively), while Braf V600E mutated melanoma cells showed almost no EdU incorporation (Figure 3A bottom). This suggests the inhibitors induced a more robust proliferation arrest in Braf-mutated cell lines. Importantly, we observed an increase in SA-β-Gal activity in Braf V600E mutated cells 9 days after BMi treatment, but not in wild-type Braf cells (Figures 3B, C).

Unlike cells treated with CP and XRA, those subjected to BMi did not show an increase in DDR foci (Figures 3D, E) or IL8 secretion (Figure 3F). In fact, when probing the SASP from a broader point of view using multiplex ELISA, BMi-treated cells exhibited a reduction in different SASP factors compare to untreated cells (Figure 3F; S2.C - G). Alternatively, in general, BMi-treated cells displayed increased levels of Bcl-2 or Bcl-XL anti-apoptotic proteins, consistent with senescence-associated apoptotic resistance (Supplementary Figures S4A, B). In summary these observations suggest that long-term Braf and Mek inhibition induces a senescent-like state in Braf V600E mutated melanoma cells, which is different from that induced by genotoxic stresses.

**FIGURE 4:**
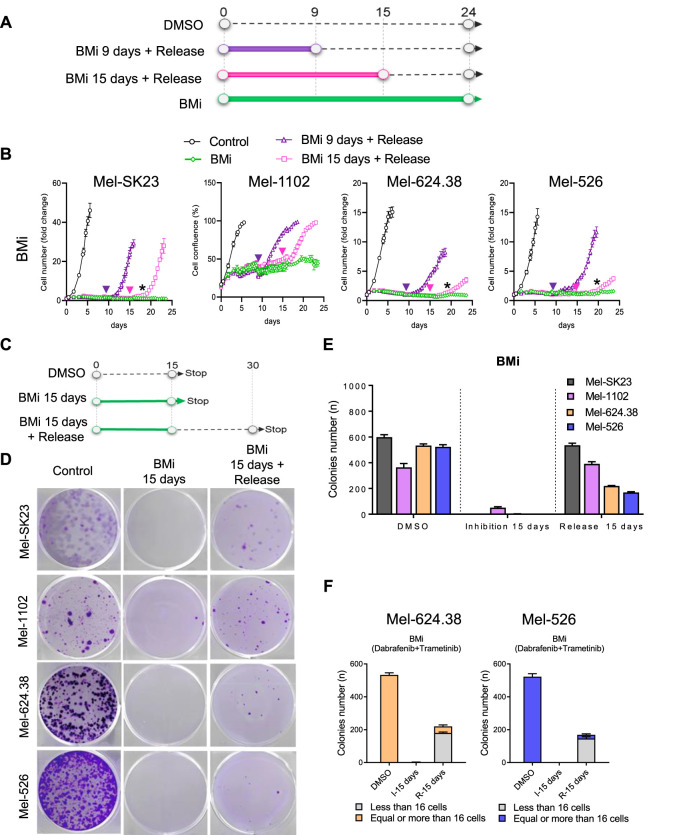
BMi-induced mixed persister cells and senescence-like phenotypes in Braf V600E melanoma cells. **(A)** Experimental outline and **(B)** proliferation curves of BMi treatment with or without release at the indicated time point across four distinct melanoma cell lines. The arrows indicate the release time for corresponding time point; the black star indicates the moment when cells resume proliferation after 15 days of BMi treatment with release. **(C)** Timeline of the colonies formation assay and **(D)** the accompanying representative images at 15 days post-BMi treatment, and after 15 days of BMi treatment followed by a 15-day release. **(E)** Histogram illustrating the number of colonies from the images presented in **(D)**. **(F)** Another histogram breaking down the number of colonies from the images in D into two size categories: those with fewer than 16 cells and those with more than 16 cells. The data shown represents the mean ± SD of triplicates and is representative of three independent experiments.

While senescence in normal cells is defined by a stable proliferation arrest, recent research in cancer cells, including Olaparib-induced senescence in high-grade serous ovarian cancer or androgen depletion-induced senescence in prostate cancer, has demonstrated the potential reversibility of a cancer senescence-like state (Fleury et al., 2019; Malaquin et al., 2020). Thus, we explored whether the senescence-like state induced by BMi is autonomously stable or require sustained inhibition of proliferation signalling. Through real-time imaging, we performed BMi treatment and release assays and observed that at least a fraction of wild-type Braf cells recovered from a BMi treatment that lasted nine or 15 days (Figure 4A). In contrast, while Braf V600E mutated cells also recovered, they experienced a significant delay compared to wild-type cells, suggesting that perhaps less cells recovered (Figure 4B).

To ascertain if recovery was widespread or limited to a subset of cells, we conducted a colony formation assay with 15 days of BMi treatment followed by a 15-day drug-free period (Figure 4C). Most wild-type Braf melanoma cells regained their proliferative capacity post-drug release (Figures 4D, E). In contrast, only approximately 30% of Braf-mutated cells regained their proliferative capacity, forming small colonies mainly consisting of 16 cells or less (Figure 4F). The cells that retain the ability to recover and proliferate may represent previously described persister cells, which can adapt to treatment (Marine et al., 2020; Chauvistre et al., 2022). Alternatively, the cell population that remain incapable of proliferation after drug release appears senescent-like, possibly via epigenetic modifications induced by prolonged Braf and Mek inhibition, which converted the majority of Braf V600E melanoma cells into a stable proliferation arrest state resembling senescence (Crouch et al., 2022). Overall, these observations support a mixed cell fate decision model in melanoma cells harboring the Braf V600E mutation in response to long-term Braf and Mek inhibition, characterized by both senescent-like cells that cannot recover after drug withdrawal (cannot form colonies), and cells that exhibit a persister phenotype (cells that maintain the ability to recover proliferation after treatment withdrawal).

### 3.4 DNA damage-induced senescence promotes melanoma cell sensitivity to Bcl2/Bcl-XL inhibitors

Senescent cells often display senescence-associated apoptosis resistance targetable using senolytics, which encompass molecules/drugs that selectively kill senescent cells without harming non-senescent cells (Chang et al., 2016; Zhu et al., 2016; Hickson et al., 2019). Sensitivity to senolytics, particularly Bcl2-family inhibitors, varies based on cancer type and senescence inducer (Fleury et al., 2019; Malaquin et al., 2020; Lafontaine et al., 2021). Considering the distinct melanoma cell fate phenotypes induced by genotoxic stresses or BMi, we evaluated the levels of the anti-apoptotic proteins Bcl-2 or Bcl-XL. In general, both proteins were detected even in untreated cells, and at least one of these proteins was upregulated in senescent-ike BMi-induced cells consistent with the potential induction of Bcl2-family apoptotic resistance in this context. However, this was not the case during CP-induced senescence, where only p53 wild-type mel-SK23 and mel-1102 showed increased levels (Supplementary Figures S4A, B).

**FIGURE 5:**
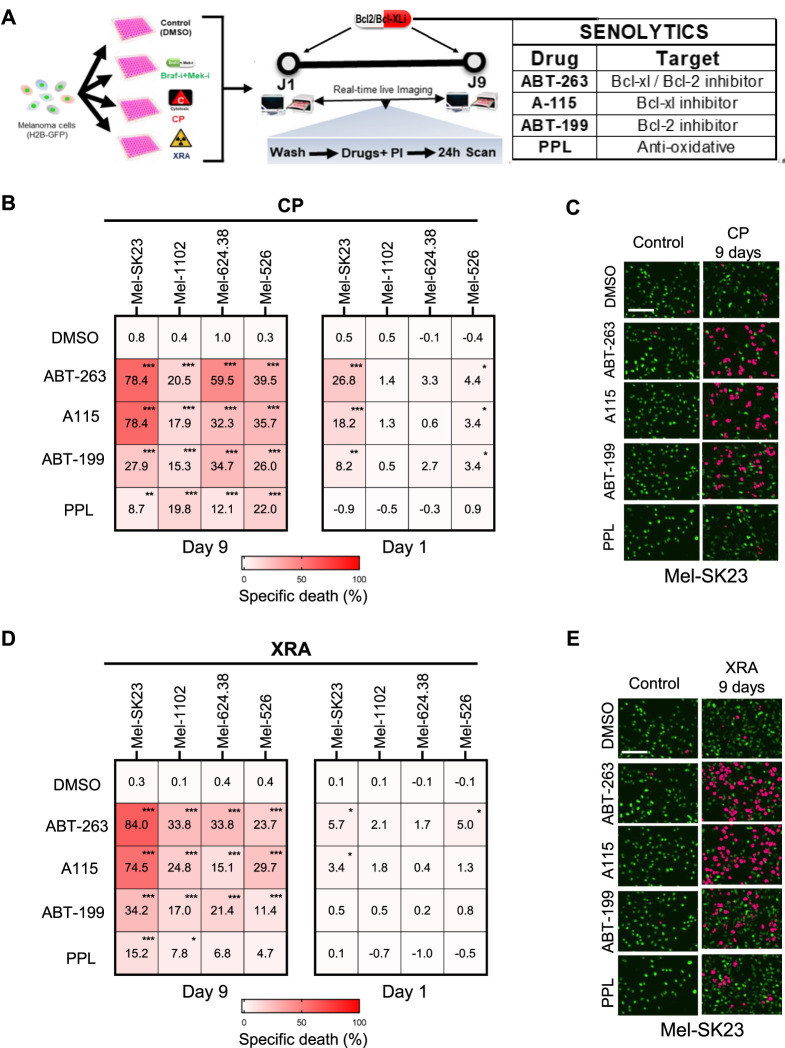
DNA damage-induced senescence sensitizes melanoma cells to Bcl2/Bcl-XL inhibitors. **(A)** Experimental design for the senolytic assay. The pre-treatment conditions are shown on the left, while the senolytic assay on the right (Day 1 or Day 9 following pre-treatment) involves washing cells with PBS and adding fresh culture media containing PI and senolytic compounds, followed by live imaging over 24 h **(B,C)** Heat maps illustrating the percent of specific death **(B)** and complemented by representative images **(C)**, captured 24 h after treatment with senolytics (ABT263–0.32µM; A115–0,32 μM; ABT199 1.25 µM; or PPL - 0.32µM) in the four melanoma cell lines that were initially pre-treated with CP for 1 or 9 days. **(D,E)** Heat maps showing the percentage of specific cell death **(D)** and accompanied by representative images **(E)**, captured 24 h after treatment with senolytics (ABT263–0.32 µM; A115–0,32 μM; ABT199 1.25 µM; or PPL - 0.32 µM) in the four melanoma cell lines that were initially exposed to XRA for 1- or 9-days. The percentage of specific cell death were calculated using chromium-51 assay formula. Data represents the mean ± SD of triplicates and is representative of three independent experiments, with statistical significance indicated by * for *p* < 0.05, ** for *p* < 0.01, and *** for *p* < 0.001, as determined by two-way ANOVA followed by Dunnett’s multiple comparisons test.

To directly test senolytic sensitivity, we first determined the IC50 of the tested senolytic drugs for each cell line (Supplementary Figure S1A) and employed a real-time death assay to test cell sensitivity to senolytic drugs (at a lower dose than the IC50), including: ABT-263, Bcl2 and Bcl-XL inhibitor (Wendt, 2008), A-115 (Bcl-XL inhibitor) (Tao et al., 2014), ABT-199 (Bcl2 inhibitor) (Chang et al., 2016), and Piperlongumine (PPL, a natural antioxidant product) (Wang et al., 2016). CP and XRA-induced senescent melanoma cells demonstrated significant sensitivity to these senolytics, particularly ABT-263, A-115, and ABT-199, which were more effective than PPL in inducing cell death (Figure 5. B left, C, D left, E). This suggests that both Bcl-XL and Bcl2 play critical roles in the survival of senescent melanoma cells induced by CP or XRA, regardless of whether they are induced or not during senescence (Supplementary Figures S4A, B).

To confirm the specificity of the senolytic effect, we repeated the assay 1 day after CP or XRA treatment (Figures 5B, D, right). Early after treatment, three out of four cell lines were no longer or less sensitive to these drugs, indicating that the initial sensitivity of CP or XRA-treated cells primarily arises from their senescence state, not a direct synergy with DNA damage or CP. As an exception, Mel-SK23 cells were strongly sensitive to Bcl2/Bcl-XL inhibitors immediately after CP exposure, suggesting that these cells are sensitive to Bcl2 inhibitors-induced apoptosis when exposed to DNA damage, even before senescence. Overall, these findings suggest that Bcl2/Bcl-XL inhibitors can induce cell death in DNA damage-induced senescent melanoma cells.

### 3.5 Direct synergy between combo BMi and Bcl2/Bcl-XL inhibitors promote melanoma cells death in TIS independent manner

**FIGURE 6:**
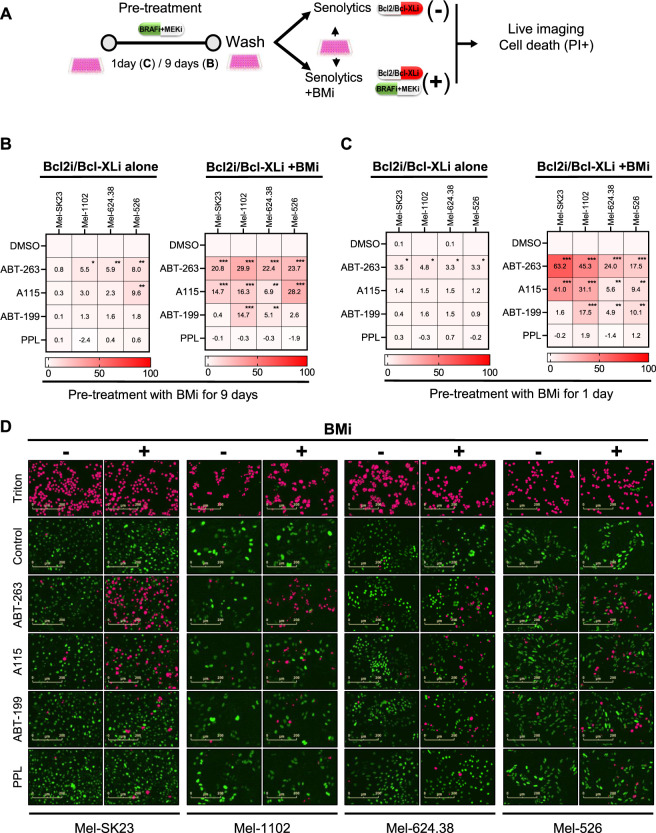
Direct synergy between BMi and Bcl2/Bcl-XL inhibitors promote melanoma cells death in a cell fate independent manner. **(A)** Experimental design for assessing drug synergy based on specific cell death. Prior to the synergy assessment, cells were initially pretreated with BMi for 1 or 9 days. **(B,C)** Heat maps illustrating the percentage of specific cell death 24 h after treatment with senolytics alone (Bcl2i/Bcl-XLi alone) or in combination with BMi (Bcl2i/Bcl-XLi + BMi) in different melanoma cell lines. These cells were pretreated with BMi for 9 days **(B)** or 1 day **(C)**. **(D)** Representative images of the data presented in C, with H2BGFP count in green and a mask of PI/H2B-GFP double positive count in pink. Data shown represents the mean ± SD of triplicates and is representative of three independent experiments, with statistical significance indicated by * for *p* < 0.05, ** for *p* < 0.01, and *** for *p* < 0.001, as determined by two-way ANOVA followed by Dunnett’s multiple comparisons test.

We then investigated the sensitivity of BMi-induced phenotypes to senolytics. Given the reported direct synthetic lethality between Mek and Bcl2/Bcl-XL inhibitors (Cragg et al., 2008; Corcoran et al., 2013; Iavarone et al., 2019) and the mixed nature of BMi-induced phenotypes (Figure 4B), we used a sequential treatment approach with or without BMi (Figure 6A).

Unlike CP and XRA-induced senescent cells, BMi-induced senescent-like and persister cells showed no or less sensitivity to Bcl2/Bcl-XL inhibitors or PPL when treated after induction of senescence and removal of BMi (Figure 6B left). However, simultaneous treatment with senolytics and BMi led to significant inscrease in cell death, especially with Bcl2/Bcl-XL inhibitors (Figure 6B right). This indicates that a direct synergy between Bcl2/Bcl-XL inhibitors and BMi, rather than with BMi-induced senescence phenotypes, triggers melanoma cell death (Airiau et al., 2016).

To confirm direct BMi-Bcl2 synergy, we conducted another cell death assay 1 day after BMi treatment (Figure 6C). We added senolytics with or without BMi. The simultaneous combination of Bcl2/Bcl-XL inhibitors and BMi induced melanoma cell death (Figure 6C right), unlike Bcl2/Bcl-XL inhibitors alone (Figure 6C left). These results show that the BMi-induced senescence-like or persister state does not sensitize melanoma cells to Bcl2/Bcl-XL inhibitors and PPL. However, as previously reported, a direct cooperation between Bcl2/Bcl-XL inhibitors and BMi used simultaneously provoke both non-senescent and senescent melanoma cell death.

## 4 Discussion

This study delve into the impact of various therapies on melanoma cell fate outcomes, with a primary focus on therapy-induced senescence and the potential for combining these treatments with senolytic drugs. Senescence is a complex cellular response, acting both as a beneficial mechanism by limiting the proliferation of damaged cells and a potentially detrimental one by promoting the proliferation of neighboring non-senescent cells (Sun et al., 2018). Our findings shed light on how melanoma cells respond to clinically relevant therapies (CP, XRA, and BMi) and the diverse phenotypes they exhibit. These insights hold implications for designing more effective melanoma treatment strategies.

BMi effectively halted MAP-kinase signaling and specifically restrained the proliferation of Braf mutant melanoma cells, aligning with clinical responses in patients with Braf mutations (Chapman et al., 2011). This underscores the significance of personalized treatment based on tumor genetics. Nevertheless, the limited responsiveness of NRAS-mutated melanoma cells to BMi combination therapy, primarily due to paradoxical ERK activation in response to RAF inhibitors, represents a challenge in the design of targeted therapies including those that perhaps induce senescence (Lai et al., 2022). The observed p90-RSK phosphorylation in Mel-1102 cells following BMi treatment is consistent with this idea, indicating a compensatory mechanism leading to reduced proliferation inhibition when both drugs are combined (Supplementary Figure S1B). This phenomenon has significant implications for treatment efficacy, resistance mechanisms and necessitates the exploration of alternative treatments for this patient subset.

Our observations revealed that CP and XRA treatments elicited mixed responses, encompassing cell death and cellular senescence, regardless of BRAF mutation profiles. This aligns with prior studies demonstrating the common occurrence of cellular senescence in melanoma cells exposed to cisplatin-based treatments (Sun et al., 2018), primarily mediated through the DNA damage response (DDR) and the p53/p21 axis. In cases of p53 mutant cells, the involvement of alternate mediators like EZH2 might explain p21 expression, considering EZH2’s repressive effect on p21/CDKN1A via interaction with HDAC1 (Fan et al., 2011). During genotoxic-induced melanoma senescence, p53-independent p21/CDKN1A expression may result from EZH2 phosphorylation and ATM-mediated degradation (Ito et al., 2018). Notably, Mel-624.38 cells did not display any p21 or p16 upregulation (Supplementary Figures S4A, B), revealing that other pathways in melanoma cells can potentially generate a senescence-like cell cycle arrest. In general, genotoxic treatment-induced senescence in melanoma generates a stable senescence state including a SASP, emphasizing the potential implication of these cells for the tumor microenvironment and therapeutic targeting.

Long-term BMi treatment leads to mixed population phenotypes encompassing senescent-like cells and persister cells in Braf V600E melanoma cells. Persister cells, which retain the ability to recover and proliferate after treatment withdrawal, contrast with the traditionally autonomously stable arrest associated with cellular senescence (Hayflick and Moorhead, 1961). This adaptability suggests a survival strategy that allows these cells to withstand therapeutic pressure (Hangauer et al., 2017).

The remaining senescent-like cells exhibited stable G1 proliferation arrest and increased SA-β-Gal activity but did not display elevated DDR foci, SASP, or Bcl2-family senolytic sensitivity. This aligns with prior reports indicating that BMi induce senescence-like in BRAF mutant melanoma cells, often accompanied by autophagy, G1 arrest, p27KIP1 induction, and pRb activation (Paraiso et al., 2010; Haferkamp et al., 2013; Schick et al., 2015; Li et al., 2016; Krayem et al., 2018; Madorsky Rowdo et al., 2020). Notably, the G1 arrest was transient for persister cells, as they regained proliferative capacity after BMi washout, possibly due to rapid phospho-ERK recovery, a known mechanism for therapy escape (Paraiso et al., 2010). Autophagy, one key feature of persister cells (Chauvistre et al., 2022), might also contribute to their reversibility. Unlike senescent-like cells, persister cells do not necessarily exhibit a complete senescence program but are characterized by their ability to evade therapeutic pressures and contribute to tumor recurrence upon treatment withdrawal (Zhao et al., 2023). As another potential explanation, prolonged BRAF and MEK inhibition (8 days or more) can lead to senescence via Myc degradation and ERBB3 upregulation, linked to primary resistance to MAPK-targeted therapies (Sun et al., 2014; Hayes et al., 2016).

Extended BRAF + MEK inhibition resulted in a delayed return of cell proliferation after drug removal, possibly due to induced epigenetic changes in the enduring senescence-like state. This state, which showed resistance to Bcl2-family senolytics, can be affected by epigenetic inhibitors like HDACi and CDK9i, inducing cell death (Madorsky Rowdo et al., 2020). The observations support the notion that long-term BRAF and MEK inhibition can induce a senescence-like phenotype with unique epigenetic modifications. This distinct behavior in Braf-mutated cells underscores that treatment response depends on both cell type and the specific modality, emphasizing the importance of considering genetic alterations in designing targeted senescence therapies.

We investigated the cooperative effect of Bcl2/Bcl-XL inhibitors with BMi in melanoma cell viability. These inhibitors displayed sensitivity when used alongside BMi, irrespective of cell fate. Similar synergistic effect has been observed in various contexts, inducing apoptosis in diverse cancer models like KRAS mutant cancer (Corcoran et al., 2013; Koyama et al., 2020), non-small cell lung cancer (Tan et al., 2013) and high-grade serous ovarian cancer patient-derived xenograft (Iavarone et al., 2019). The MAPK pathway, activated in BRAF V600E mutation melanomas, regulates apoptosis through effectors such as BAD and BIM. MEK inhibition enhances Bcl2/Bcl-XL inhibitors’ cytotoxicity by promoting BIM dephosphorylation and binding to MCL-1 (Korfi et al., 2016). Our findings confirm the direct synergy between BRAF + MEK and Bcl2/Bcl-XL inhibitors, emphasizing simultaneous inhibition’s feasibility and reducing the interest in a sequential approach in the context of senescence therapy.

In conclusion, CP and XRA effectively induce senescence in melanoma cells through DDR, enhancing their vulnerability to Bcl2/Bcl-XL inhibitors. On the other hand, BMi triggers a mixed cell fate decision including senescence-like and persister state that cannot be targeted via sequential senolytics approaches, potentially contributing to relapse and resistance. Further *in vivo* studies are needed for validation. Combining senolytics with traditional treatments or BMi shows promise for selectively eliminating senescent cells. Senotherapeutics could benefit treatment-resistant or secondary melanoma patients. Investigating the interplay between TIS and the adaptive immune system, particularly tumor-infiltrating lymphocytes (TILs), holds relevance in diverse therapeutic contexts for melanoma and other cancers.

## Data Availability

The original contributions presented in the study are included in the article/Supplementary Material, further inquiries can be directed to the corresponding author.

## References

[B1] Airiau K. Prouzet-Mauleon V. Rousseau B. Pigneux A. Jeanneteau M. Giraudon M. (2016). Synergistic cooperation between ABT-263 and MEK1/2 inhibitor: effect on apoptosis and proliferation of acute myeloid leukemia cells. Oncotarget 7, 845–859. 10.18632/oncotarget.6417 26625317 PMC4808037

[B2] Baar M. P. Brandt R. M. C. Putavet D. A. Klein J. D. D. Derks K. W. J. Bourgeois B. R. M. (2017). Targeted apoptosis of senescent cells restores tissue homeostasis in response to chemotoxicity and aging. Cell 169, 132–147. 10.1016/j.cell.2017.02.031 28340339 PMC5556182

[B3] Balakrishnan I. Danis E. Pierce A. Madhavan K. Wang D. Dahl N. (2020). Senescence induced by BMI1 inhibition is a therapeutic vulnerability in H3K27m-mutant DIPG. Cell Rep. 33, 108286. 10.1016/j.celrep.2020.108286 33086074 PMC7574900

[B4] Belloc F. Dumain P. Boisseau M. R. Jalloustre C. Reiffers J. Bernard P. (1994). A flow cytometric method using Hoechst 33342 and propidium iodide for simultaneous cell cycle analysis and apoptosis determination in unfixed cells. Cytometry 17, 59–65. 10.1002/cyto.990170108 7528124

[B5] Bray F. Ferlay J. Soerjomataram I. Siegel R. L. Torre L. A. Jemal A. (2018). Global cancer statistics 2018: GLOBOCAN estimates of incidence and mortality worldwide for 36 cancers in 185 countries. CA Cancer J. Clin. 68, 394–424. 10.3322/caac.21492 30207593

[B6] Chakrabarty A. Chakraborty S. Bhattacharya R. Chowdhury G. (2021). Senescence-induced chemoresistance in triple negative breast cancer and evolution-based treatment strategies. Front. Oncol. 11, 674354. 10.3389/fonc.2021.674354 34249714 PMC8264500

[B7] Chang J. Wang Y. Shao L. Laberge R. M. Demaria M. Campisi J. (2016). Clearance of senescent cells by ABT263 rejuvenates aged hematopoietic stem cells in mice. Nat. Med. 22, 78–83. 10.1038/nm.4010 26657143 PMC4762215

[B8] Chapman P. B. Hauschild A. Robert C. Haanen J. B. Ascierto P. Larkin J. (2011). Improved survival with vemurafenib in melanoma with BRAF V600E mutation. N. Engl. J. Med. 364, 2507–2516. 10.1056/NEJMoa1103782 21639808 PMC3549296

[B9] Chauvistre H. Shannan B. Daignault-Mill S. M. Ju R. J. Picard D. Egetemaier S. (2022). Persister state-directed transitioning and vulnerability in melanoma. Nat. Commun. 13, 3055. 10.1038/s41467-022-30641-9 35650266 PMC9160289

[B10] Corcoran R. B. Cheng K. A. Hata A. N. Faber A. C. Ebi H. Coffee E. M. (2013). Synthetic lethal interaction of combined BCL-XL and MEK inhibition promotes tumor regressions in KRAS mutant cancer models. Cancer Cell 23, 121–128. 10.1016/j.ccr.2012.11.007 23245996 PMC3667614

[B11] Cragg M. S. Jansen E. S. Cook M. Harris C. Strasser A. Scott C. L. (2008). Treatment of B-RAF mutant human tumor cells with a MEK inhibitor requires Bim and is enhanced by a BH3 mimetic. J. Clin. Invest. 118, 3651–3659. 10.1172/JCI35437 18949058 PMC2571034

[B12] Crouch J. Shvedova M. Thanapaul R. Botchkarev V. Roh D. (2022). Epigenetic regulation of cellular senescence. Cells 11, 672. 10.3390/cells11040672 35203320 PMC8870565

[B13] Cummings B. S. Schnellmann R. G. (2004). Measurement of cell death in mammalian cells. Curr. Protoc. Pharmacol. Chapter 12, Unit 12 8. 10.1002/0471141755.ph1208s25 PMC3874588 22294120

[B14] Davies H. Bignell G. R. Cox C. Stephens P. Edkins S. Clegg S. (2002). Mutations of the BRAF gene in human cancer. Nature 417, 949–954. 10.1038/nature00766 12068308

[B15] Debacq-Chainiaux F. Erusalimsky J. D. Campisi J. Toussaint O. (2009). Protocols to detect senescence-associated beta-galactosidase (SA-betagal) activity, a biomarker of senescent cells in culture and *in vivo* . Nat. Protoc. 4, 1798–1806. 10.1038/nprot.2009.191 20010931

[B16] Edlundh-Rose E. Egyhazi S. Omholt K. Mansson-Brahme E. Platz A. Hansson J. (2006). NRAS and BRAF mutations in melanoma tumours in relation to clinical characteristics: a study based on mutation screening by pyrosequencing. Melanoma Res. 16, 471–478. 10.1097/01.cmr.0000232300.22032.86 17119447

[B17] Elliott C. L. Allport V. C. Loudon J. A. Wu G. D. Bennett P. R. (2001). Nuclear factor-kappa B is essential for up-regulation of interleukin-8 expression in human amnion and cervical epithelial cells. Mol. Hum. Reprod. 7, 787–790. 10.1093/molehr/7.8.787 11470867

[B18] Fan T. Jiang S. Chung N. Alikhan A. Ni C. Lee C. C. (2011). EZH2-dependent suppression of a cellular senescence phenotype in melanoma cells by inhibition of p21/CDKN1A expression. Mol. Cancer Res. 9, 418–429. 10.1158/1541-7786.MCR-10-0511 21383005 PMC3078218

[B19] Ferlay J. Colombet M. Soerjomataram I. Parkin D. M. Pineros M. Znaor A. (2021). Estimating the global cancer incidence and mortality in 2018: GLOBOCAN sources and methods. Int. J. Cancer 144, 1941–1953. 10.1002/ijc.31937 30350310

[B20] Filimon A. Preda I. A. Boloca A. F. Negroiu G. (2021). Interleukin-8 in melanoma pathogenesis, prognosis and therapy-an integrated view into other neoplasms and chemokine networks. Cells 11, 120. 10.3390/cells11010120 35011682 PMC8750532

[B21] Flaherty K. T. Robert C. Hersey P. Nathan P. Garbe C. Milhem M. (2012). Improved survival with MEK inhibition in BRAF-mutated melanoma. N. Engl. J. Med. 367, 107–114. 10.1056/NEJMoa1203421 22663011

[B22] Fleury H. Malaquin N. Tu V. Gilbert S. Martinez A. Olivier M. A. (2019). Exploiting interconnected synthetic lethal interactions between PARP inhibition and cancer cell reversible senescence. Nat. Commun. 10, 2556. 10.1038/s41467-019-10460-1 31186408 PMC6560032

[B23] Fu X. Li M. Tang C. Huang Z. Najafi M. (2021). Targeting of cancer cell death mechanisms by resveratrol: a review. Apoptosis 26, 561–573. 10.1007/s10495-021-01689-7 34561763

[B24] Ghadaouia S. Olivier M. A. Martinez A. Kientega T. Qin J. Lambert-Lanteigne P. (2021). Homologous recombination-mediated irreversible genome damage underlies telomere-induced senescence. Nucleic Acids Res. 49, 11690–11707. 10.1093/nar/gkab965 34725692 PMC8599762

[B25] Giuliano S. Ohanna M. Ballotti R. Bertolotto C. (2011). Advances in melanoma senescence and potential clinical application. Pigment. Cell Melanoma Res. 24, 295–308. 10.1111/j.1755-148X.2010.00820.x 21143770

[B26] Goldwirt L. Louveau B. Baroudjian B. Allayous C. Jouenne F. Da Meda L. (2021). Dabrafenib and trametinib exposure-efficacy and tolerance in metastatic melanoma patients: a pharmacokinetic-pharmacodynamic real-life study. Cancer Chemother. Pharmacol. 88, 427–437. 10.1007/s00280-021-04299-x 34057572

[B27] Guillon J. Petit C. Toutain B. Guette C. Lelievre E. Coqueret O. (2019). Chemotherapy-induced senescence, an adaptive mechanism driving resistance and tumor heterogeneity. Cell Cycle 18, 2385–2397. 10.1080/15384101.2019.1652047 31397193 PMC6738909

[B28] Haferkamp S. Borst A. Adam C. Becker T. M. Motschenbacher S. Windhovel S. (2013). Vemurafenib induces senescence features in melanoma cells. J. Invest. Dermatol 133, 1601–1609. 10.1038/jid.2013.6 23321925

[B29] Hangauer M. J. Viswanathan V. S. Ryan M. J. Bole D. Eaton J. K. Matov A. (2017). Drug-tolerant persister cancer cells are vulnerable to GPX4 inhibition. Nature 551, 247–250. 10.1038/nature24297 29088702 PMC5933935

[B30] Havel J. J. Chowell D. Chan T. A. (2019). The evolving landscape of biomarkers for checkpoint inhibitor immunotherapy. Nat. Rev. Cancer 19, 133–150. 10.1038/s41568-019-0116-x 30755690 PMC6705396

[B31] Hayes T. K. Neel N. F. Hu C. Gautam P. Chenard M. Long B. (2016). Long-term ERK inhibition in KRAS-mutant pancreatic cancer is associated with MYC degradation and senescence-like growth suppression. Cancer Cell 29, 75–89. 10.1016/j.ccell.2015.11.011 26725216 PMC4816652

[B32] Hayflick L. (1965). The limited *in vitro* lifetime of human diploid cell strains. Exp. Cell Res. 37, 614–636. 10.1016/0014-4827(65)90211-9 14315085

[B33] Hayflick L. Moorhead P. S. (1961). The serial cultivation of human diploid cell strains. Exp. Cell Res. 25, 585–621. 10.1016/0014-4827(61)90192-6 13905658

[B34] Hickson L. J. Langhi Prata L. G. P. Bobart S. A. Evans T. K. Giorgadze N. Hashmi S. K. (2019). Senolytics decrease senescent cells in humans: preliminary report from a clinical trial of Dasatinib plus Quercetin in individuals with diabetic kidney disease. EBioMedicine 47, 446–456. 10.1016/j.ebiom.2019.08.069 31542391 PMC6796530

[B35] Hodis E. Watson I. R. Kryukov G. V. Arold S. T. Imielinski M. Theurillat J. P. (2012). A landscape of driver mutations in melanoma. Cell 150, 251–263. 10.1016/j.cell.2012.06.024 22817889 PMC3600117

[B36] Huizing M. T. Giaccone G. van Warmerdam L. J. Rosing H. Bakker P. J. Vermorken J. B. (1997). Pharmacokinetics of paclitaxel and carboplatin in a dose-escalating and dose-sequencing study in patients with non-small-cell lung cancer. The European Cancer Centre. J. Clin. Oncol. 15, 317–329. 10.1200/JCO.1997.15.1.317 8996159

[B37] Iavarone C. Zervantonakis I. K. Selfors L. M. Palakurthi S. Liu J. F. Drapkin R. (2019). Combined MEK and BCL-2/XL inhibition is effective in high-grade serous ovarian cancer patient-derived xenograft models and BIM levels are predictive of responsiveness. Mol. Cancer Ther. 18, 642–655. 10.1158/1535-7163.MCT-18-0413 30679390 PMC6399746

[B38] Ito T. Teo Y. V. Evans S. A. Neretti N. Sedivy J. M. (2018). Regulation of cellular senescence by polycomb chromatin modifiers through distinct DNA damage- and histone methylation-dependent pathways. Cell Rep. 22, 3480–3492. 10.1016/j.celrep.2018.03.002 29590617 PMC5915310

[B39] Jaber S. Warnier M. Leers C. Vernier M. Goehrig D. Medard J. J. (2023). Targeting chemoresistant senescent pancreatic cancer cells improves conventional treatment efficacy. Mol. Biomed. 4, 4. 10.1186/s43556-023-00116-4 36739330 PMC9899302

[B40] Jochems F. Thijssen B. De Conti G. Jansen R. Pogacar Z. Groot K. (2021). The Cancer SENESCopedia: a delineation of cancer cell senescence. Cell Rep. 36, 109441. 10.1016/j.celrep.2021.109441 34320349 PMC8333195

[B41] Jost T. Heinzerling L. Fietkau R. Hecht M. Distel L. V. (2021). Palbociclib induces senescence in melanoma and breast cancer cells and leads to additive growth arrest in combination with irradiation. Front. Oncol. 11, 740002. 10.3389/fonc.2021.740002 34722291 PMC8548830

[B42] Korfi K. Smith M. Swan J. Somervaille T. C. Dhomen N. Marais R. (2016). BIM mediates synergistic killing of B-cell acute lymphoblastic leukemia cells by BCL-2 and MEK inhibitors. Cell Death Dis. 7, e2177. 10.1038/cddis.2016.70 27054332 PMC4855656

[B43] Koyama M. Kitazawa M. Nakamura S. Matsumura T. Miyazaki S. Miyagawa Y. (2020). Low-dose trametinib and Bcl-xL antagonist have a specific antitumor effect in KRAS-mutated colorectal cancer cells. Int. J. Oncol. 57, 1179–1191. 10.3892/ijo.2020.5117 32901840

[B44] Krayem M. Najem A. Journe F. Morandini R. Sales F. Awada A. (2018). Acquired resistance to BRAFi reverses senescence-like phenotype in mutant BRAF melanoma. Oncotarget 9, 31888–31903. 10.18632/oncotarget.25879 30159130 PMC6112757

[B45] Lafontaine J. Cardin G. B. Malaquin N. Boisvert J. S. Rodier F. Wong P. (2021). Senolytic targeting of bcl-2 anti-apoptotic family increases cell death in irradiated sarcoma cells. Cancers (Basel) 13, 386. 10.3390/cancers13030386 33494434 PMC7866159

[B46] Lai L. P. Fer N. Burgan W. Wall V. E. Xu B. Soppet D. (2022). Classical RAS proteins are not essential for paradoxical ERK activation induced by RAF inhibitors. Proc. Natl. Acad. Sci. U. S. A. 119, e2113491119. 10.1073/pnas.2113491119 35091470 PMC8812530

[B47] Li Z. Jiang K. Zhu X. Lin G. Song F. Zhao Y. (2016). Encorafenib (LGX818), a potent BRAF inhibitor, induces senescence accompanied by autophagy in BRAFV600E melanoma cells. Cancer Lett. 370, 332–344. 10.1016/j.canlet.2015.11.015 26586345

[B48] Liang X. Lin X. Lin Z. Lin W. Peng Z. Wei S. (2023). Genes associated with cellular senescence favor melanoma prognosis by stimulating immune responses in tumor microenvironment. Comput. Biol. Med. 158, 106850. 10.1016/j.compbiomed.2023.106850 37031510

[B49] Lin K. Zhou Y. Lin Y. Feng Y. Chen Y. Cai L. (2023). Senescence-related lncRNA signature predicts prognosis, response to immunotherapy and chemotherapy in skin cutaneous melanoma. Biomolecules 13, 661. 10.3390/biom13040661 37189408 PMC10135470

[B50] Madorsky Rowdo F. P. Baron A. Gallagher S. J. Hersey P. Emran A. A. Von Euw E. M. (2020). Epigenetic inhibitors eliminate senescent melanoma BRAFV600E cells that survive long-term BRAF inhibition. Int. J. Oncol. 56, 1429–1441. 10.3892/ijo.2020.5031 32236593 PMC7170042

[B51] Maertens O. Johnson B. Hollstein P. Frederick D. T. Cooper Z. A. Messiaen L. (2013). Elucidating distinct roles for NF1 in melanomagenesis. Cancer Discov. 3, 338–349. 10.1158/2159-8290.CD-12-0313 23171796 PMC3595355

[B52] Malaquin N. Vancayseele A. Gilbert S. Antenor-Habazac L. Olivier M. A. Ait Ali Brahem Z. (2020). DNA damage- but not enzalutamide-induced senescence in prostate cancer promotes senolytic bcl-xL inhibitor sensitivity. Cells 9, 1593. 10.3390/cells9071593 32630281 PMC7408442

[B53] Marine J. C. Dawson S. J. Dawson M. A. (2020). Non-genetic mechanisms of therapeutic resistance in cancer. Nat. Rev. Cancer 20, 743–756. 10.1038/s41568-020-00302-4 33033407

[B54] Mhaidat N. M. Z. Allen X. D. Avery-Kiejda J. Scott K. A. Hersey R. J. Hersey P. (2007). Temozolomide induces senescence but not apoptosis in human melanoma cells. Br. J. Cancer 97, 1225–1233. 10.1038/sj.bjc.6604017 17968428 PMC2360470

[B55] Milanovic M. Fan D. N. Y. Belenki D. Dabritz J. H. M. Zhao Z. Yu Y. (2018). Senescence-associated reprogramming promotes cancer stemness. Nature 553, 96–100. 10.1038/nature25167 29258294

[B56] Mylonas K. J. O'Sullivan E. D. Humphries D. Baird D. P. Docherty M. H. Neely S. A. (2021). Cellular senescence inhibits renal regeneration after injury in mice, with senolytic treatment promoting repair. Sci. Transl. Med. 13, eabb0203. 10.1126/scitranslmed.abb0203 34011625

[B57] Oubaha M. Miloudi K. Dejda A. Guber V. Mawambo G. Germain M. A. (2016). Senescence-associated secretory phenotype contributes to pathological angiogenesis in retinopathy. Sci. Transl. Med. 8, 362ra144. 10.1126/scitranslmed.aaf9440 27797960

[B58] Ozols R. F. Bundy B. N. Greer B. E. Fowler J. M. Clarke-Pearson D. Burger R. A. (2003). Phase III trial of carboplatin and paclitaxel compared with cisplatin and paclitaxel in patients with optimally resected stage III ovarian cancer: a Gynecologic Oncology Group study. J. Clin. Oncol. 21, 3194–3200. 10.1200/JCO.2003.02.153 12860964

[B59] Paraiso K. H. Fedorenko I. V. Cantini L. P. Munko A. C. Hall M. Sondak V. K. (2010). Recovery of phospho-ERK activity allows melanoma cells to escape from BRAF inhibitor therapy. Br. J. Cancer 102, 1724–1730. 10.1038/sj.bjc.6605714 20531415 PMC2883709

[B60] Perez-Lorenzo R. Zheng B. (2012). Targeted inhibition of BRAF kinase: opportunities and challenges for therapeutics in melanoma. Biosci. Rep. 32, 25–33. 10.1042/BSR20110068 21981139 PMC3837566

[B61] Puszkiel A. Noe G. Bellesoeur A. Kramkimel N. Paludetto M. N. Thomas-Schoemann A. (2019). Clinical pharmacokinetics and pharmacodynamics of Dabrafenib. Clin. Pharmacokinet. 58, 451–467. 10.1007/s40262-018-0703-0 30094711

[B62] Rodier F. Munoz D. P. Teachenor R. Chu V. Le O. Bhaumik D. (2011). DNA-SCARS: distinct nuclear structures that sustain damage-induced senescence growth arrest and inflammatory cytokine secretion. J. Cell Sci. 124, 68–81. 10.1242/jcs.071340 21118958 PMC3001408

[B63] Ruscetti M. Morris J. P. t. Mezzadra R. Russell J. Leibold J. Romesser P. B. (2021). Senescence-induced vascular remodeling creates therapeutic vulnerabilities in pancreas cancer. Cell 184, 4838–4839. 10.1016/j.cell.2021.07.028 34478658 PMC10336720

[B64] Salama R. Sadaie M. Hoare M. Narita M. (2014). Cellular senescence and its effector programs. Genes Dev. 28, 99–114. 10.1101/gad.235184.113 24449267 PMC3909793

[B65] Schick U. Kyula J. Barker H. Patel R. Zaidi S. Gregory C. (2015). Trametinib radiosensitises RAS- and BRAF-mutated melanoma by perturbing cell cycle and inducing senescence. Radiother. Oncol. 117, 364–375. 10.1016/j.radonc.2015.06.026 26163092

[B66] Shain A. H. Bastian B. C. (2016). From melanocytes to melanomas. Nat. Rev. Cancer 16, 345–358. 10.1038/nrc.2016.37 27125352

[B67] Sharma P. Allison J. P. (2015). Immune checkpoint targeting in cancer therapy: toward combination strategies with curative potential. Cell 161, 205–214. 10.1016/j.cell.2015.03.030 25860605 PMC5905674

[B68] Siegel R. L. Miller K. D. Jemal A. (2020). Cancer statistics, 2020. CA Cancer J. Clin. 70, 7–30. 10.3322/caac.21590 31912902

[B69] Sun C. Hobor S. Bertotti A. Zecchin D. Huang S. Galimi F. (2014). Intrinsic resistance to MEK inhibition in KRAS mutant lung and colon cancer through transcriptional induction of ERBB3. Cell Rep. 7, 86–93. 10.1016/j.celrep.2014.02.045 24685132

[B70] Sun X. Shi B. Zheng H. Min L. Yang J. Li X. (2018). Senescence-associated secretory factors induced by cisplatin in melanoma cells promote non-senescent melanoma cell growth through activation of the ERK1/2-RSK1 pathway. Cell Death Dis. 9, 260. 10.1038/s41419-018-0303-9 29449532 PMC5833767

[B71] Tan N. Wong M. Nannini M. A. Hong R. Lee L. B. Price S. (2013). Bcl-2/Bcl-xL inhibition increases the efficacy of MEK inhibition alone and in combination with PI3 kinase inhibition in lung and pancreatic tumor models. Mol. Cancer Ther. 12, 853–864. 10.1158/1535-7163.MCT-12-0949 23475955

[B72] Tao Z. F. Hasvold L. Wang L. Wang X. Petros A. M. Park C. H. (2014). Discovery of a potent and selective BCL-XL inhibitor with *in vivo* activity. ACS Med. Chem. Lett. 5, 1088–1093. 10.1021/ml5001867 25313317 PMC4190639

[B73] Thompson E. L. Hu J. J. Niedernhofer L. J. (2021). The role of senescent cells in acquired drug resistance and secondary cancer in BRAFi-treated melanoma. Cancers (Basel) 13, 2241. 10.3390/cancers13092241 34066966 PMC8125319

[B74] Topalian S. L. Solomon D. Rosenberg S. A. (1989). Tumor-specific cytolysis by lymphocytes infiltrating human melanomas. J. Immunol. 142, 3714–3725. 10.4049/jimmunol.142.10.3714 2785562

[B75] Wallace D. Hildesheim A. Pinto L. A. (2004). Comparison of benchtop microplate beta counters with the traditional gamma counting method for measurement of chromium-51 release in cytotoxic assays. Clin. Diagn Lab. Immunol. 11, 255–260. 10.1128/cdli.11.2.255-260.2004 15013972 PMC371223

[B76] Wang C. Vegna S. Jin H. Benedict B. Lieftink C. Ramirez C. (2019). Inducing and exploiting vulnerabilities for the treatment of liver cancer. Nature 574, 268–272. 10.1038/s41586-019-1607-3 31578521 PMC6858884

[B77] Wang W. Mani A. M. Wu Z. H. (2017). DNA damage-induced nuclear factor-kappa B activation and its roles in cancer progression. J. Cancer Metastasis Treat. 3, 45–59. 10.20517/2394-4722.2017.03 28626800 PMC5472228

[B78] Wang Y. Chang J. Liu X. Zhang X. Zhang S. Zhang X. (2016). Discovery of piperlongumine as a potential novel lead for the development of senolytic agents. Aging (Albany NY) 8, 2915–2926. 10.18632/aging.101100 27913811 PMC5191878

[B79] Wendt M. D. (2008). Discovery of ABT-263, a Bcl-family protein inhibitor: observations on targeting a large protein-protein interaction. Expert Opin. Drug Discov. 3, 1123–1143. 10.1517/17460441.3.9.1123 23506184

[B80] Wu Z. Uhl B. Gires O. Reichel C. A. (2023). A transcriptomic pan-cancer signature for survival prognostication and prediction of immunotherapy response based on endothelial senescence. J. Biomed. Sci. 30, 21. 10.1186/s12929-023-00915-5 36978029 PMC10045484

[B81] Xu M. Pirtskhalava T. Farr J. N. Weigand B. M. Palmer A. K. Weivoda M. M. (2018). Senolytics improve physical function and increase lifespan in old age. Nat. Med. 24, 1246–1256. 10.1038/s41591-018-0092-9 29988130 PMC6082705

[B82] Yousefzadeh M. J. Zhu Y. McGowan S. J. Angelini L. Fuhrmann-Stroissnigg H. Xu M. (2018). Fisetin is a senotherapeutic that extends health and lifespan. EBioMedicine 36, 18–28. 10.1016/j.ebiom.2018.09.015 30279143 PMC6197652

[B83] Zhao Y. Lu T. Song Y. Wen Y. Deng Z. Fan J. (2023). Cancer cells enter an adaptive persistence to survive radiotherapy and repopulate tumor. Adv. Sci. (Weinh) 10, e2204177. 10.1002/advs.202204177 36658726 PMC10015890

[B84] Zhu Y. Tchkonia T. Fuhrmann-Stroissnigg H. Dai H. M. Ling Y. Y. Stout M. B. (2016). Identification of a novel senolytic agent, navitoclax, targeting the Bcl-2 family of anti-apoptotic factors. Aging Cell 15, 428–435. 10.1111/acel.12445 26711051 PMC4854923

