# Supplementary figures and images for: Defining melanoma combination therapies that provide senolytic sensitivity in human melanoma cells
Source: Front Cell Dev Biol. 2024 Jun 14;12:1368711. doi: 10.3389/fcell.2024.1368711 (PMC11211604; doi:10.3389/fcell.2024.1368711)

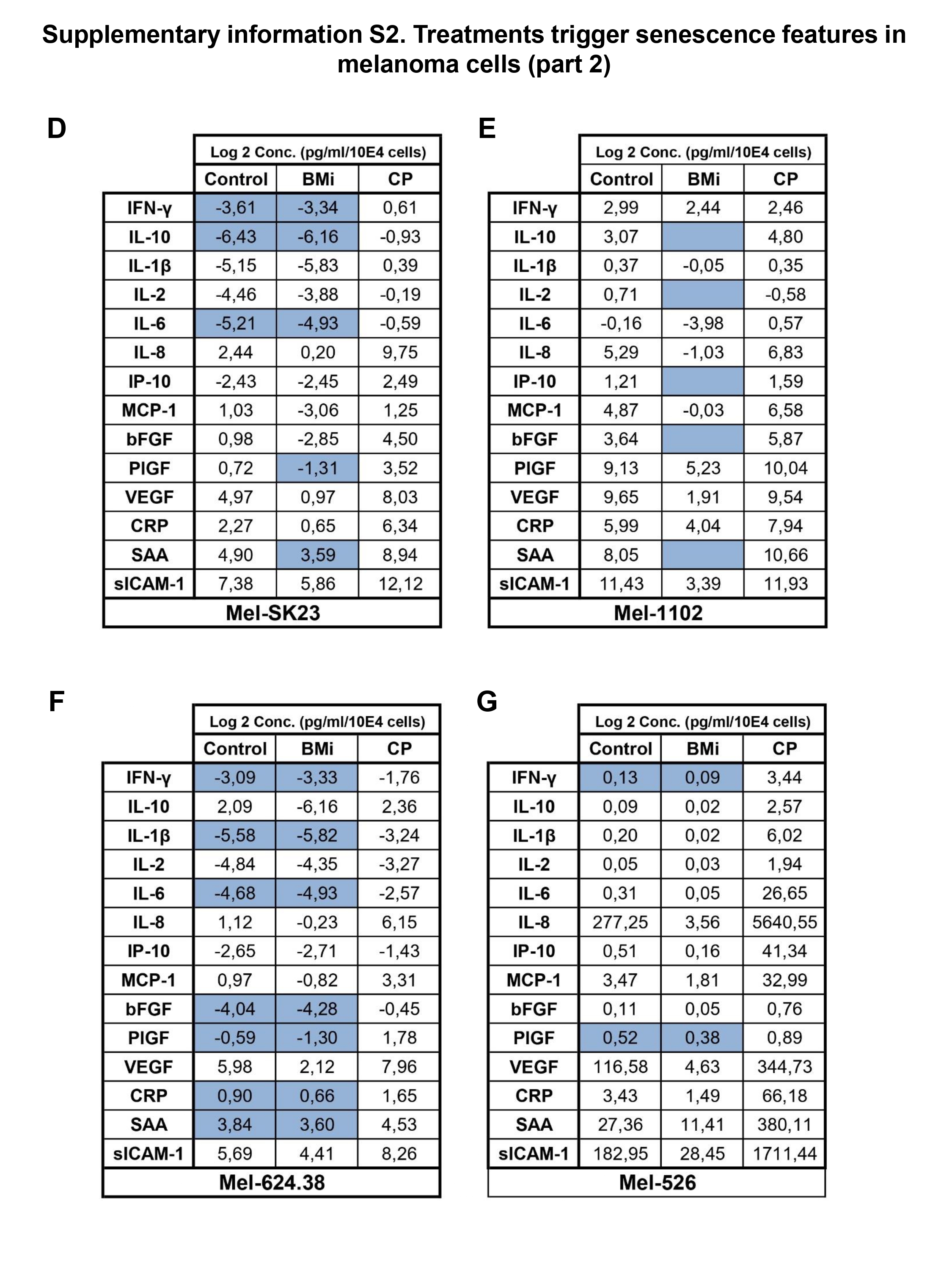

Supplement: Supplementary file 1 [file Image3.JPEG]

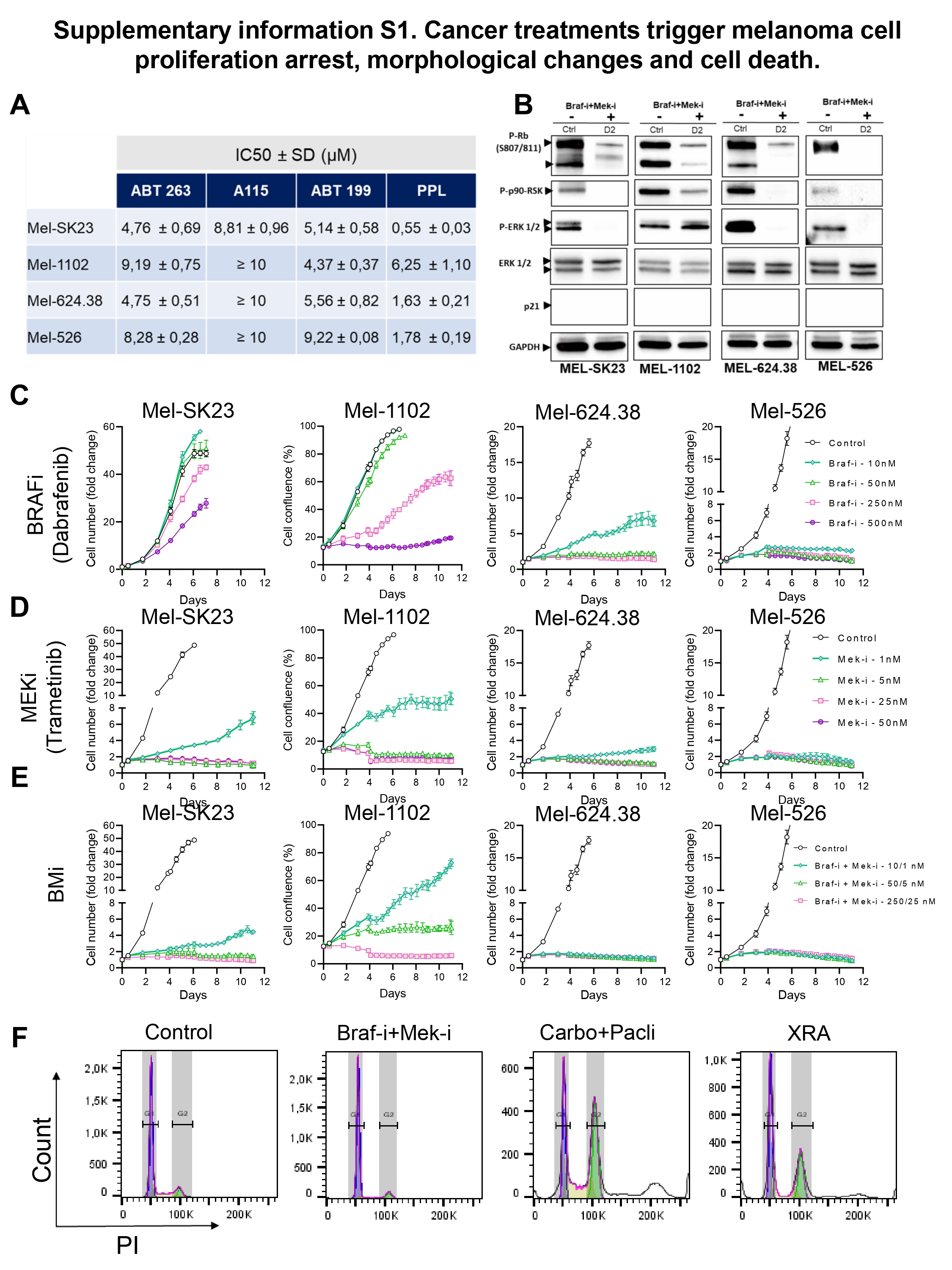

Supplement: Supplementary file 2 [file Image1.JPEG]

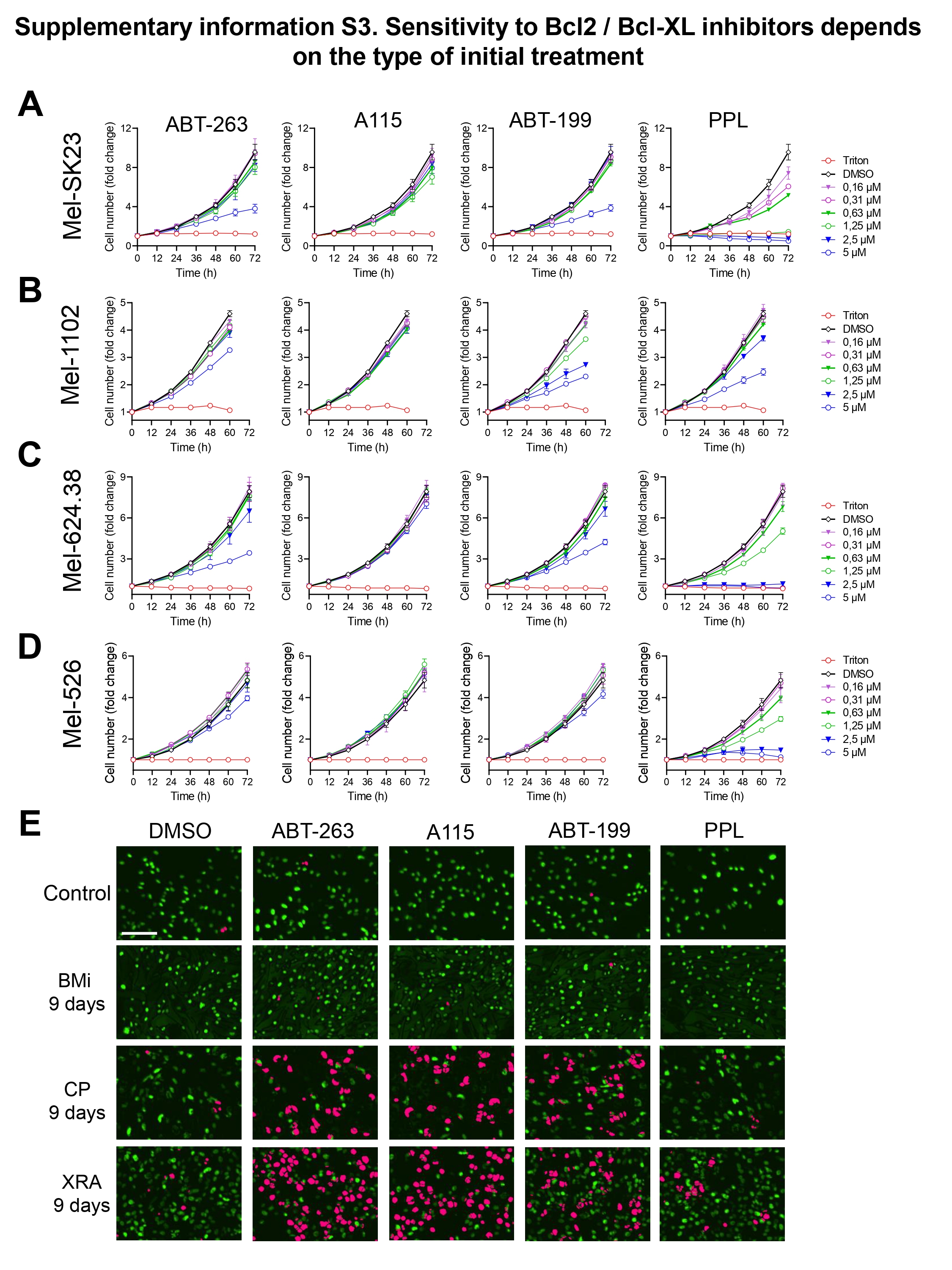

Supplement: Supplementary file 3 [file Image4.JPEG]

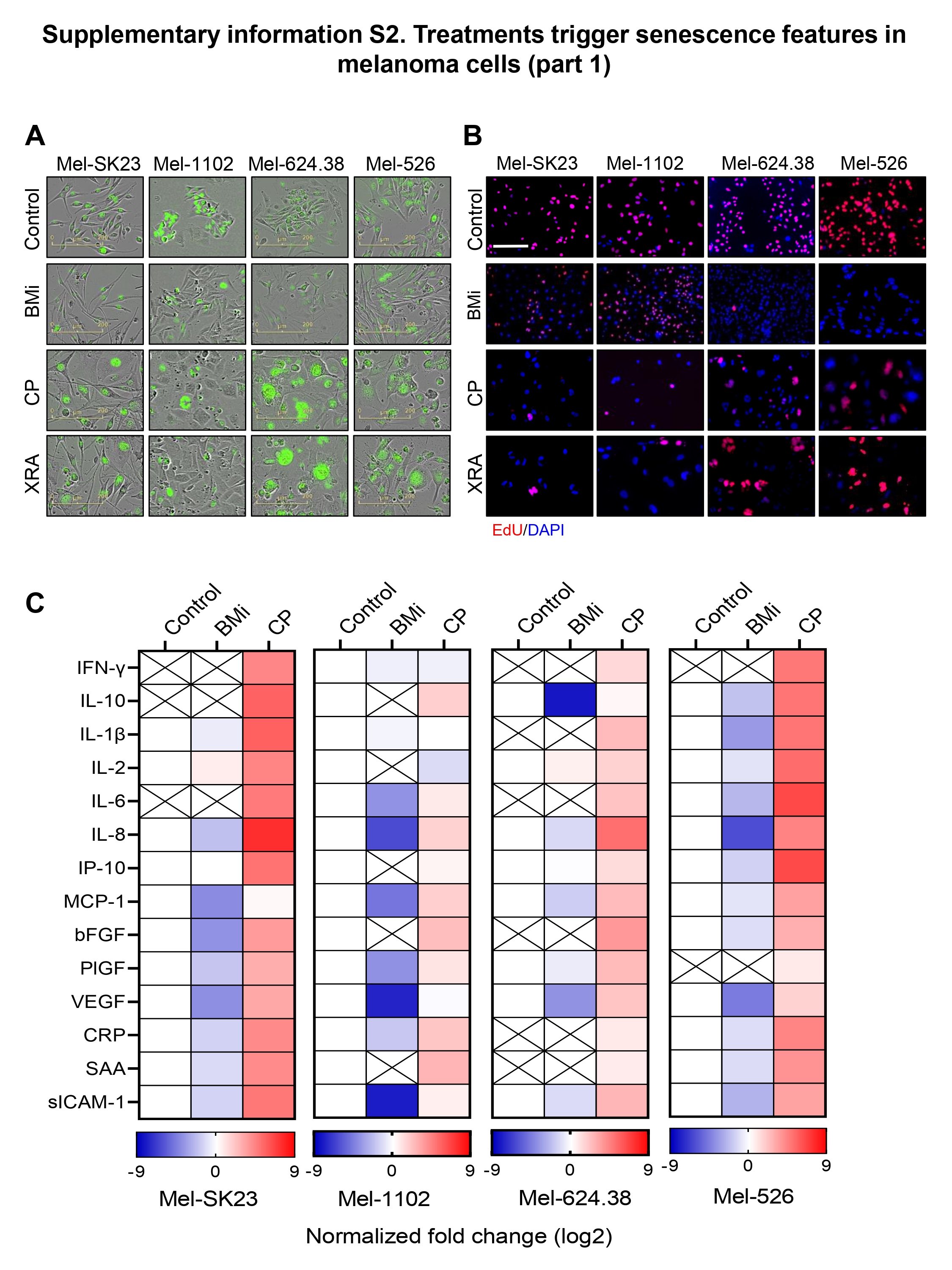

Supplement: Supplementary file 4 [file Image2.JPEG]

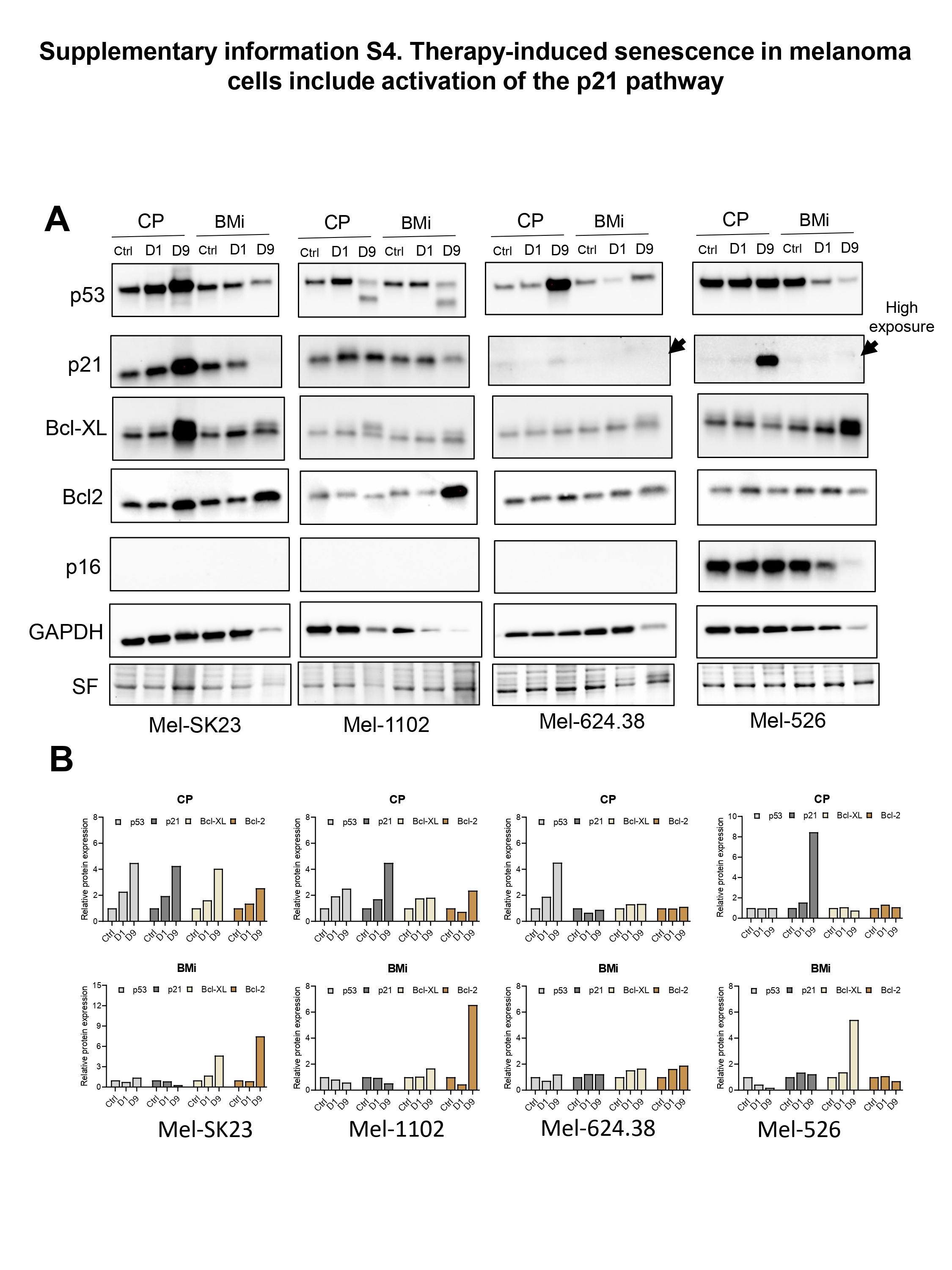

Supplement: Supplementary file 5 [file Image5.JPEG]

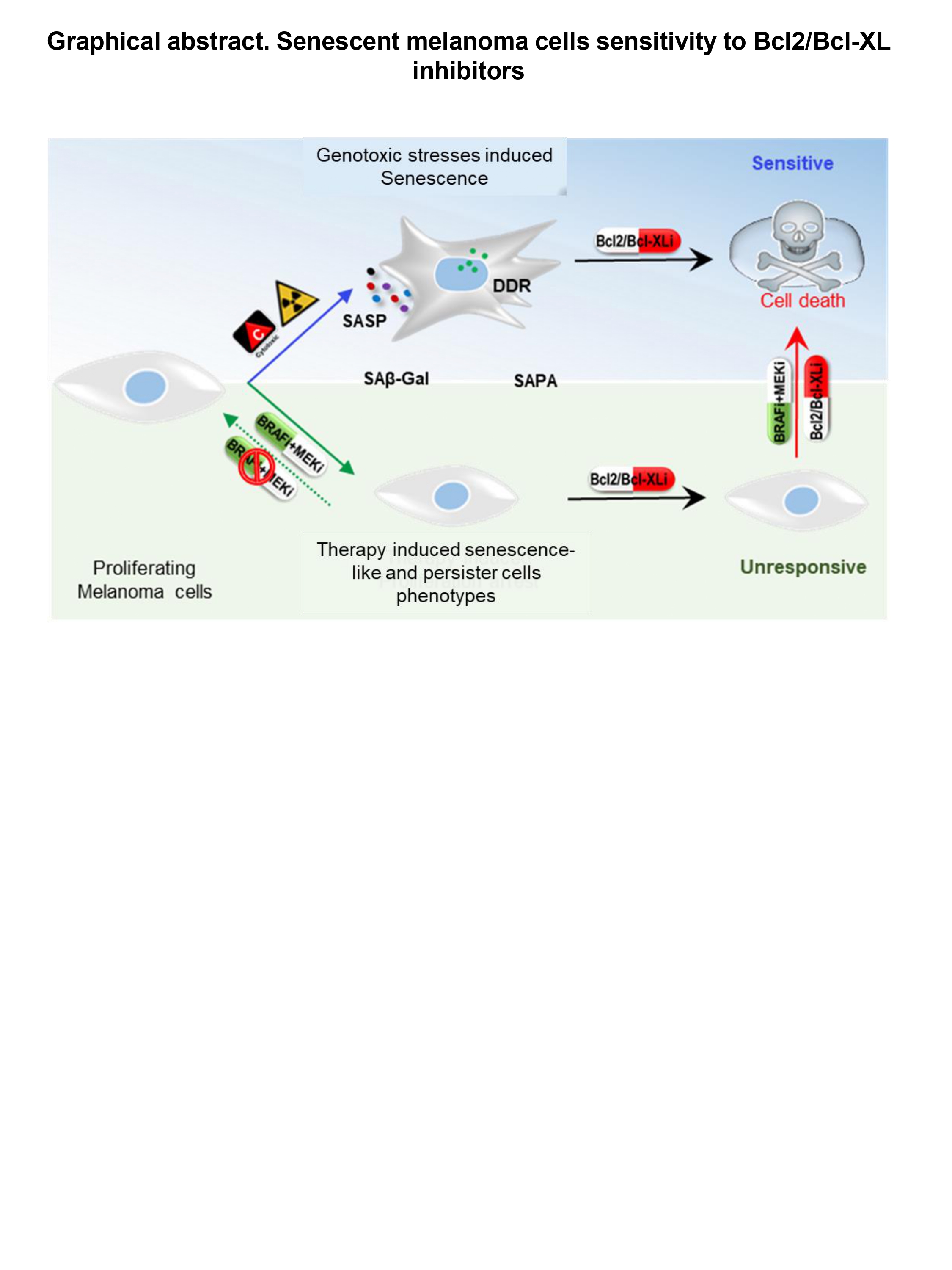

Supplement: Supplementary file 8 [file Image6.JPEG]
