# Supplementary material for: Defining melanoma combination therapies that provide senolytic sensitivity in human melanoma cells
Source: Front Cell Dev Biol. 2024 Jun 14;12:1368711. doi: 10.3389/fcell.2024.1368711 (PMC11211604; doi:10.3389/fcell.2024.1368711)

Supplementary information S1. Cancer treatments trigger melanoma cell proliferation arrest, morphological changes and cell death.

A

	IC50 ± SD (µM)			
	ABT 263	A115	ABT 199	PPL
Mel-SK23	4,76 ± 0,69	8,81 ± 0,96	5,14 ± 0,58	0,55 ± 0,03
Mel-1102	9,19 ± 0,75	≥ 10	4,37 ± 0,37	6,25 ± 1,10
Mel-624.38	4,75 ± 0,51	≥ 10	5,56 ± 0,82	1,63 ± 0,21
Mel-526	8,28 ± 0,28	≥ 10	9,22 ± 0,08	1,78 ± 0,19

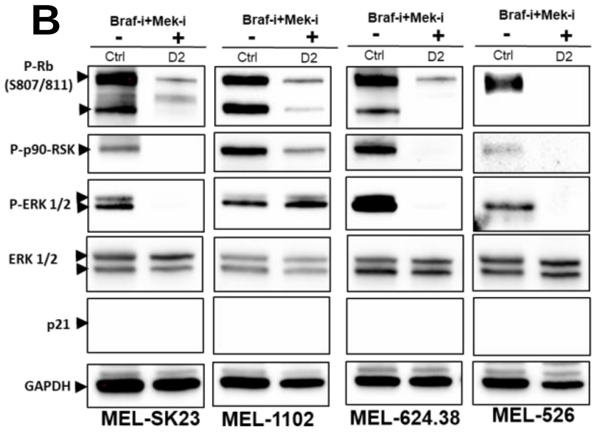

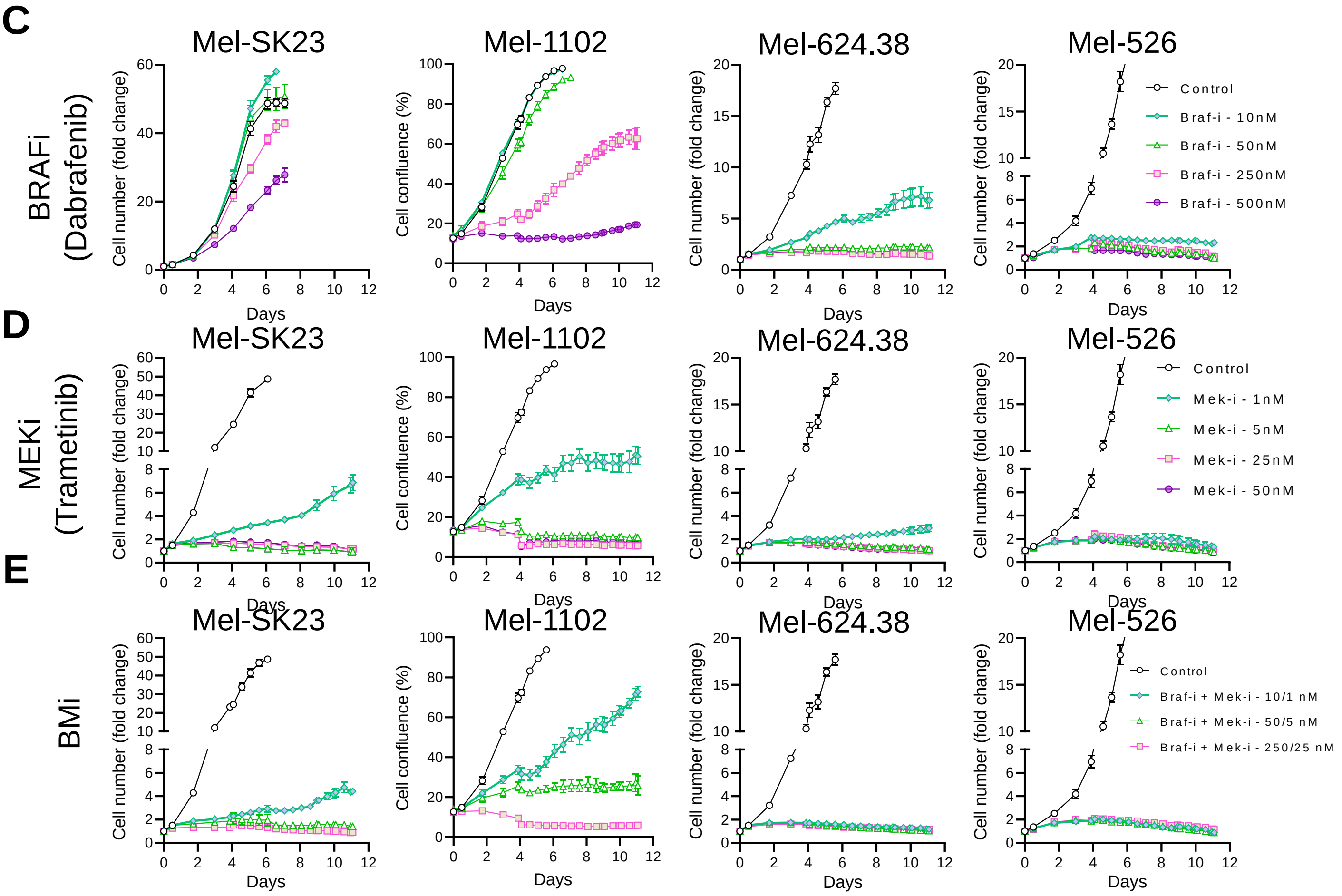

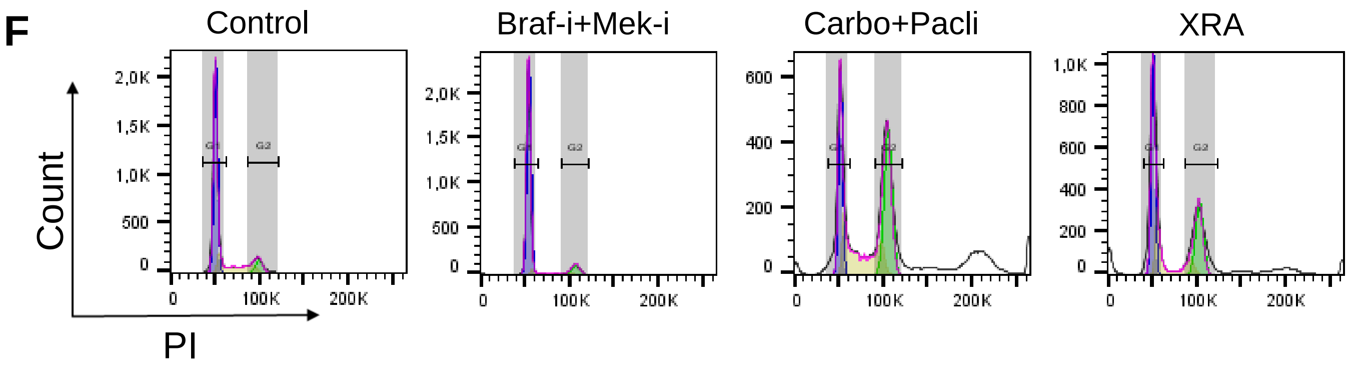

Supplementary information S2. Treatments trigger senescence features in melanoma cells (part 1)

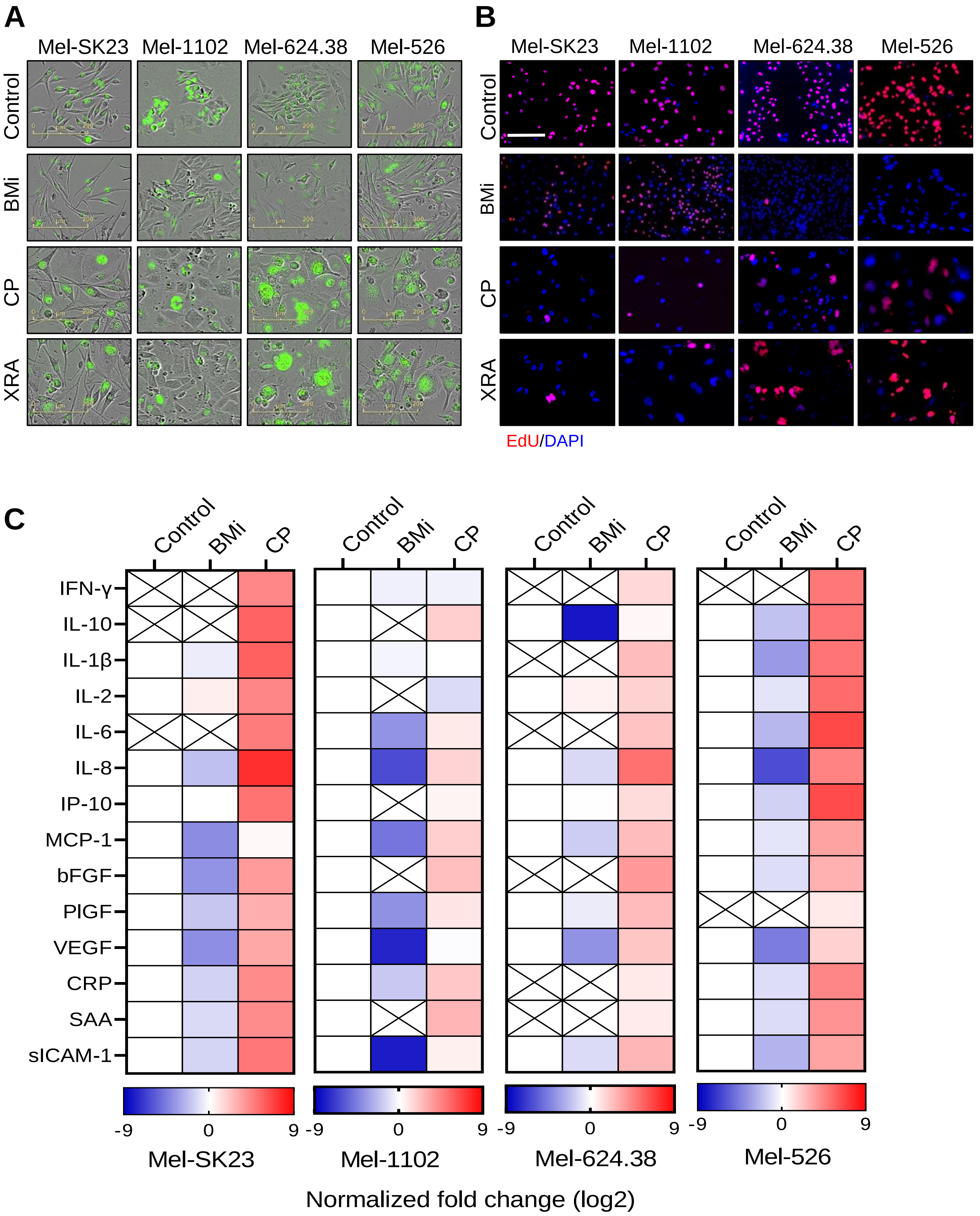

Supplementary information S2. Treatments trigger senescence features in melanoma cells (part 2)

D

	Log 2 Conc. (pg/ml/10E4 cells)		
	Control	BMi	CP
IFN-γ	-3,61	-3,34	0,61
IL-10	-6,43	-6,16	-0,93
IL-1β	-5,15	-5,83	0,39
IL-2	-4,46	-3,88	-0,19
IL-6	-5,21	-4,93	-0,59
IL-8	2,44	0,20	9,75
IP-10	-2,43	-2,45	2,49
MCP-1	1,03	-3,06	1,25
bFGF	0,98	-2,85	4,50
PIGF	0,72	-1,31	3,52
VEGF	4,97	0,97	8,03
CRP	2,27	0,65	6,34
SAA	4,90	3,59	8,94
sICAM-1	7,38	5,86	12,12
Mel-SK23			

E

	Log 2 Conc. (pg/ml/10E4 cells)		
	Control	BMi	CP
IFN-γ	2,99	2,44	2,46
IL-10	3,07		4,80
IL-1β	0,37	-0,05	0,35
IL-2	0,71		-0,58
IL-6	-0,16	-3,98	0,57
IL-8	5,29	-1,03	6,83
IP-10	1,21		1,59
MCP-1	4,87	-0,03	6,58
bFGF	3,64		5,87
PIGF	9,13	5,23	10,04
VEGF	9,65	1,91	9,54
CRP	5,99	4,04	7,94
SAA	8,05		10,66
sICAM-1	11,43	3,39	11,93
Mel-1102			

F

	Log 2 Conc. (pg/ml/10E4 cells)		
	Control	BMi	CP
IFN-γ	-3,09	-3,33	-1,76
IL-10	2,09	-6,16	2,36
IL-1β	-5,58	-5,82	-3,24
IL-2	-4,84	-4,35	-3,27
IL-6	-4,68	-4,93	-2,57
IL-8	1,12	-0,23	6,15
IP-10	-2,65	-2,71	-1,43
MCP-1	0,97	-0,82	3,31
bFGF	-4,04	-4,28	-0,45
PIGF	-0,59	-1,30	1,78
VEGF	5,98	2,12	7,96
CRP	0,90	0,66	1,65
SAA	3,84	3,60	4,53
sICAM-1	5,69	4,41	8,26
Mel-624.38			

G

	Log 2 Conc. (pg/ml/10E4 cells)		
	Control	BMi	CP
IFN-γ	0,13	0,09	3,44
IL-10	0,09	0,02	2,57
IL-1β	0,20	0,02	6,02
IL-2	0,05	0,03	1,94
IL-6	0,31	0,05	26,65
IL-8	277,25	3,56	5640,55
IP-10	0,51	0,16	41,34
MCP-1	3,47	1,81	32,99
bFGF	0,11	0,05	0,76
PIGF	0,52	0,38	0,89
VEGF	116,58	4,63	344,73
CRP	3,43	1,49	66,18
SAA	27,36	11,41	380,11
sICAM-1	182,95	28,45	1711,44
Mel-526			

Supplementary information S3. Sensitivity to Bcl2 / Bcl-XL inhibitors depends on the type of initial treatment

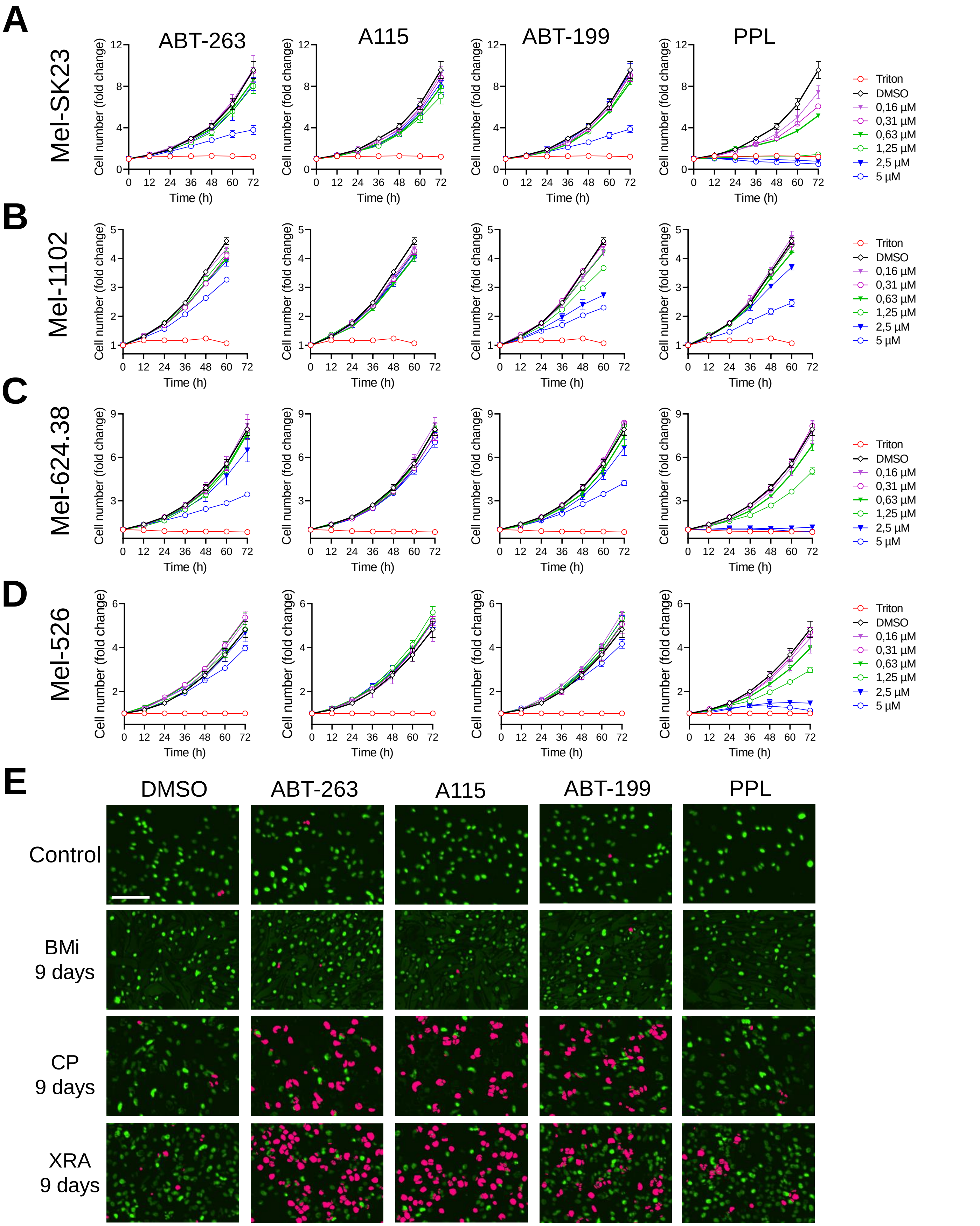

Supplementary information S4. Therapy-induced senescence in melanoma cells include activation of the p21 pathway

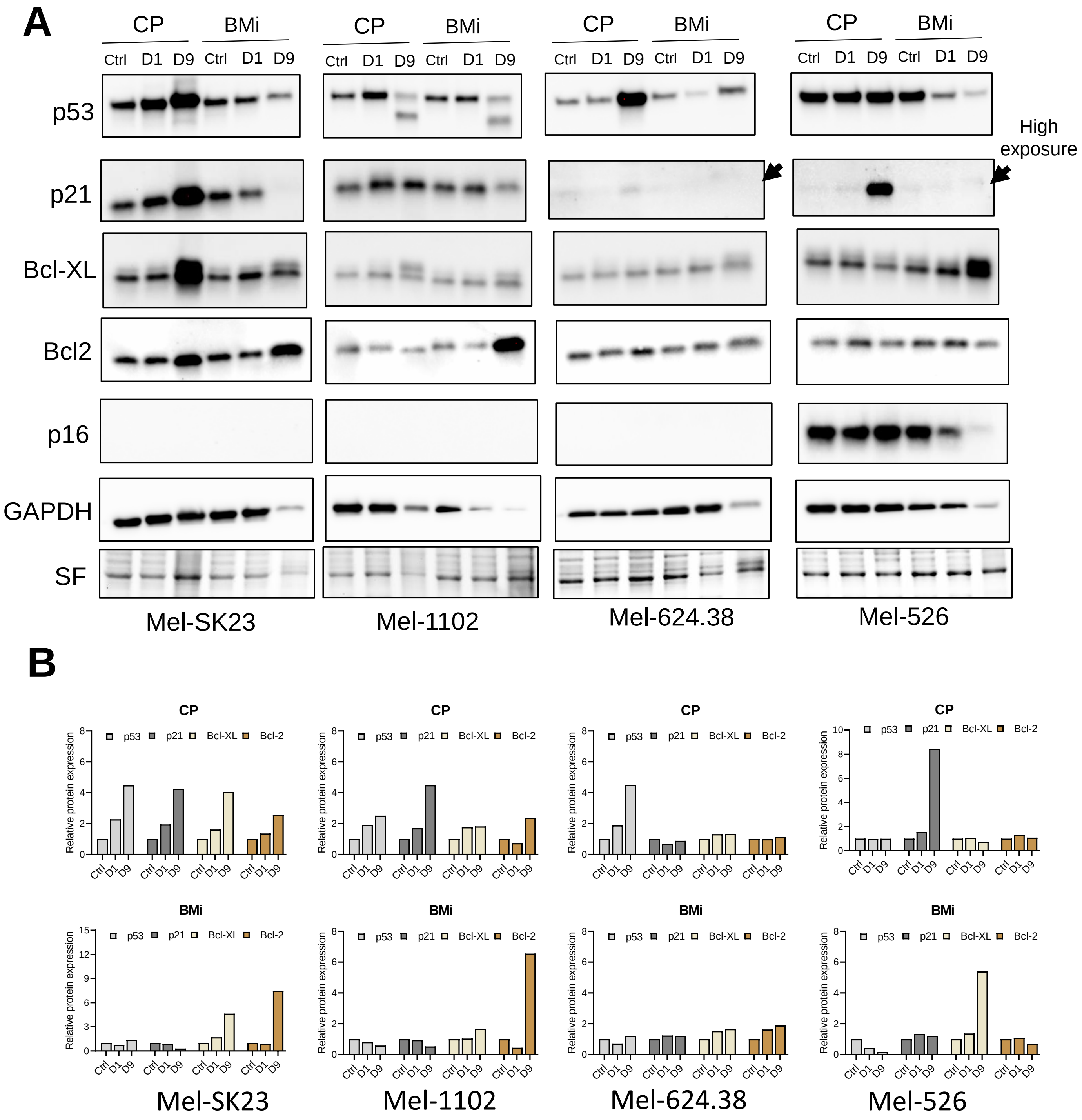

Graphical abstract. Senescent melanoma cells sensitivity to Bcl2/Bcl-XL inhibitors

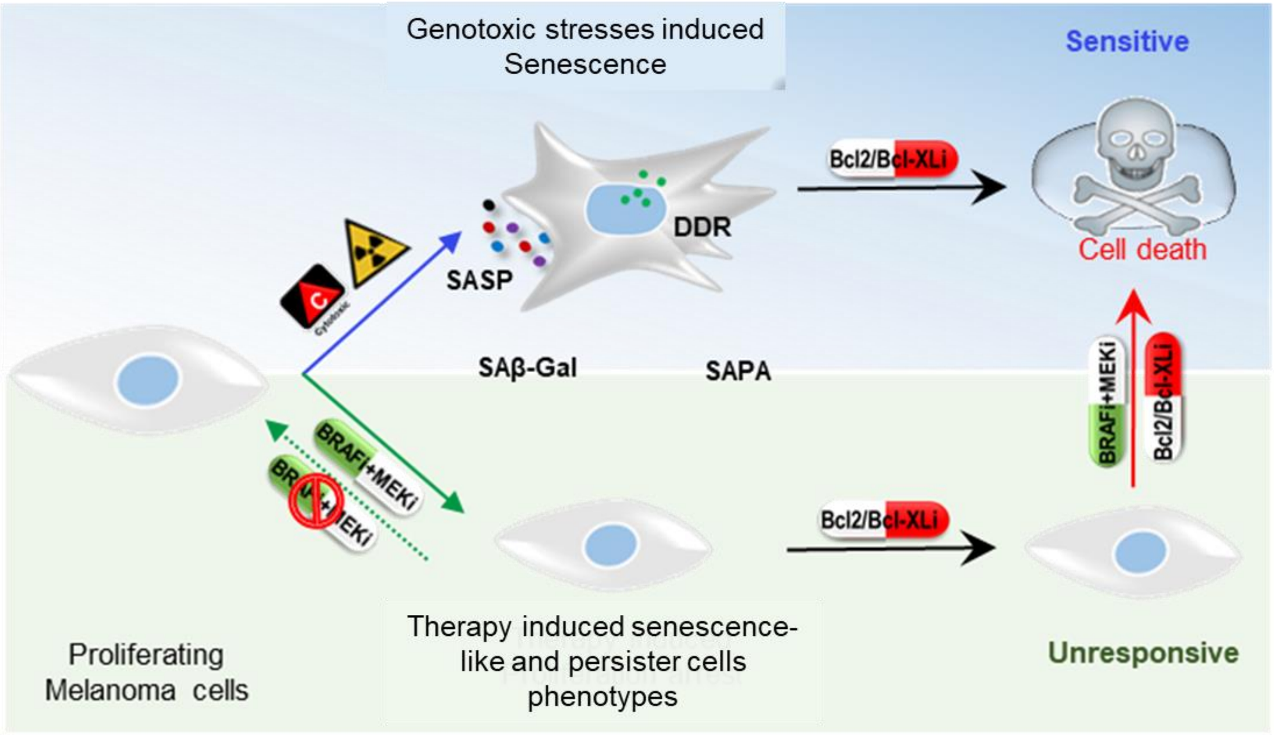

Supplement: Supplementary file 6 [file DataSheet1.PDF]
