# Supplementary material for: Defining melanoma combination therapies that provide senolytic sensitivity in human melanoma cells
Source: Front Cell Dev Biol. 2024 Jun 14;12:1368711. doi: 10.3389/fcell.2024.1368711 (PMC11211604; doi:10.3389/fcell.2024.1368711)
Supplement: Supplementary file 7 [file DataSheet1.docx]

# Supplementary information 1: Cancer treatments trigger melanoma cell proliferation arrest, morphological changes, and cell death.

**A**) Table of the IC50 values of melanoma cells in response to various tested senolytic compounds. **B)** Immunoblotting demonstrate the inhibition of downstream molecules in the MAPK/ERK pathway by BMi following 48 hours of inhibition. **C-E)** Proliferation curves depict the response of melanoma cells to different doses of: **C)** Dabrafenib (Braf-i), **D)** Trametinib (MEKi), and **E)** the combination of BMi. **F**) Flow cytometric analysis of the distribution of cell cycle phases in Mel-1102 cells, nine days after the indicated treatments. The data is presented as Mean ± SD and is representative of at least 2 independent experiments.

# Supplementary information 2: Treatments trigger senescence features in melanoma cells.

**A**) Representative images of melanoma cells expressing H2B-GFP, showing their untreated state and the state on day 6 following each treatment. **B**) Representative images illustrating EdU pulse assay conducted from day 8 to day 9 post-treatment. (C-G) Cells were either untreated or exposed to CP or BMi treatement for 9 days, and CM were collected for the following 48h. Secreted soluble factors were evaluated using a multiplex ELISA (40-Plex-MSD). C) Average secretion of untreated cells was used as baseline. Heatmap indicating log2- fold change of 14 secreted factors from control, or tables indicating log2 concentration (pg/ml/10E4 cells) of 14 secreted factors from Mel-SK23, Mel-1102, Mel-624.38 and Mel-526 cell lines (D, E, F and G respectively). Blue highlights indicate values below detection range or nor detected. Data are representative of two independent experiments.

# Supplementary information 3: Sensitivity to Bcl2/Bcl-XL inhibitors depends on the type of initial treatment

**(A-D)** Proliferation curves for **A)** Mel-SK23**,** **B)** Mel-1102, **C)** Mel-624.38 and **D)** Mel-526 cells, when exposed to a panel of four senolytic drugs (ABT-263, A115, ABT-199 and PPL) at varying dose ranges from 0.16µM to 5µM. **E)** Representative images illustrating cell death (depicted as H2BGFP in green and the PI/H2B-GFP double positive cells in pink) occurring 24 hours after the application of senolytic treatments. The senolytic drugs employed include ABT-263, a Bcl2/Bcl-XL inhibitor; ABT-199, a Bcl2 inhibitor; A-115, a Bcl-XL inhibitor; and PPL, an antioxidant. Data are representative of 3 independent experiments.

# Supplementary information 4: Therapy-induced senescence in melanoma cells include activation of the p21 pathway.

A) Western blot analysis and B) corresponding quantification normalized to stain free (SF) blot showing the expression levels of p53, p21, Bcl-XL and Bcl-2 at various time points in different melanoma cell lines when exposed to either CP or BMi. Data are representative of two independent experiments. Bcl-2 and Bcl-XL were detected in one experiment.

# Graphical abstract:

Illustration of the differential response of melanoma cells to various treatments and the potential for senolytic drugs to selectively induce death in senescent melanoma cells. In brief, proliferating melanoma cells (left) are subjected to some form of therapy that causes diverse cell responses (blue and green arrows). Cells could enter a state of senescence or therapy induced persister cells and senescence-like phenotypes (middle). On the top (left), genotoxic stresses triggers a senescent state, characterized by DDR (DNA Damage Response) and SASP (Senescence-Associated Secretory Phenotype) as well as SA-β-Gal. On the bottom (left), BRAF and MEK inhibitors (BMi) triggers a persister state (reversible, dotted green arrow) and senescence like state (in BRAF mutated cells).

On the right side, we see two distinct outcomes: cells rendered senescent by genotoxic stresses respond to Bcl2/Bcl-XL inhibitors, resulting in cell death (top right). Conversely, cells undergoing BMi -induced persister or senescence-like phenotypes show no response to Bcl2/Bcl-XL inhibitors and remain unresponsive (bottom right). However, these cells can be induced to die by a concurrent combination of BMi and Bcl2/Bcl-XL inhibitors (red arrow).
